# Reddit language indicates changes associated with diet, physical activity, substance use, and smoking during COVID-19

**DOI:** 10.1371/journal.pone.0280337

**Published:** 2023-02-03

**Authors:** Karan Wanchoo, Matthew Abrams, Raina M. Merchant, Lyle Ungar, Sharath Chandra Guntuku

**Affiliations:** 1 Department of Electrical and Systems Engineering, University of Pennsylvania, Philadelphia, Pennsylvania, United States of America; 2 Penn Medicine Center for Digital Health, University of Pennsylvania, Philadelphia, Pennsylvania, United States of America; 3 Department of Computer and Information Science, University of Pennsylvania, Philadelphia, Pennsylvania, United States of America; University of California San Diego School of Medicine, UNITED STATES

## Abstract

COVID-19 has adversely impacted the health behaviors of billions of people across the globe, modifying their former trends in health and lifestyle. In this paper, we compare the psychosocial language markers associated with diet, physical activity, substance use, and smoking before and after the onset of COVID-19 pandemic. We leverage the popular social media platform Reddit to analyze 1 million posts between January 6, 2019, to January 5, 2021, from 22 different communities (i.e., subreddits) that belong to four broader groups—diet, physical activity, substance use, and smoking. We identified that before the COVID-19 pandemic, posts involved sharing information about vacation, international travel, work, family, consumption of illicit substances, vaping, and alcohol, whereas during the pandemic, posts contained emotional content associated with quarantine, withdrawal symptoms, anxiety, attempts to quit smoking, cravings, weight loss, and physical fitness. Prevalent topic analysis showed that the pandemic was associated with discussions about nutrition, physical fitness, and outdoor activities such as backpacking and biking, suggesting users’ focus shifted toward their physical health during the pandemic. Starting from the week of March 23, 2020, when several stay-at-home policies were enacted, users wrote more about coping with stress and anxiety, alcohol misuse and abuse, and harm-reduction strategies like switching from hard liquor to beer/wine after people were socially isolated. In addition, posts related to use of substances such as benzodiazepines (valium, xanax, clonazepam), nootropics (kratom, phenibut), and opioids peaked around March 23, 2020, followed by a decline. Of note, unlike the general decline observed, the volume of posts related to alternatives to heroin (e.g., fentanyl) increased during the COVID-19 pandemic. Posts about quitting smoking gained momentum after late March 2020, and there was a sharp decline in posts about craving to smoke. This study highlights the significance of studying social media discussions on platforms like Reddit which are a rich ecological source of human experiences and provide insights to inform targeted messaging and mitigation strategies, and further complement ongoing traditional primary data collection methods.

## 1. Introduction

With the COVID-19 outbreak in early 2020, people struggled with uncertainty and social isolation and experienced loss of income, mobility, social engagement [1] and collectively these factors negatively impacted physical health [2].

Several recent studies aimed to observe and quantify the impact of COVID-19 on physical health using different empirical methods. A prospective study aimed at assessing the impact of pandemic restrictions on physical activity patterns of Dutch patients affected with cardiovascular disease (CVD) found a minor increase in physical activity levels versus a larger increase in sedentary time in Dutch CVD patients during the first wave of COVID-19 pandemic [2]. Online questionnaires measuring physical activity, physical and mental health, anxiety, and depressive symptoms in France and Switzerland also report a significant increase in sedentary behavior in the initial weeks of pandemic [3]. A recent study conducted in the United States found that the blood pressure of both men and women increased significantly from April 2020 through December 2020, compared with the same period in 2019 [4].

In this study, we sought to evaluate the expression of health and lifestyle behavior changes surrounding diet, physical activity, substance use, and smoking as the COVID-19 pandemic unfolded. People are increasingly using social media to post and share about their health and lifestyle behaviors [5–7]. We use natural language processing and machine learning techniques on text conversations extracted from a popular publicly available social media platform, Reddit, to evaluate the impact of COVID-19 on health and lifestyle behaviors that are known risk factors of cardiovascular disease [8]. Our fundamental hypothesis was that the health behaviors depicted by language of people active on Reddit in the specified subreddits would be significantly different after the pandemic struck the world. This language would reveal how health behavior choices of individuals changed pre-pandemic versus during pandemic.

### 1.1 Background and related work

#### 1.1.1 Cardiovascular risk factors and COVID-19

Several mortality studies showed a clear increase in coronary heart disease mortality with an increase in the number of cigarettes smoked per day, regardless of the actual number [9, 10]. Further, it has been shown that both active and passive smoking contribute to more than 30% of coronary heart disease (CHD) mortality cases [11]. Alcohol consumption of three or more drinks is associated with increased blood pressure and triglycerides levels [12]. In another study, light to moderate alcohol intake was associated with 51% CVD mortality compared with abstainers [13]. Similarly, diet and physical activity contribute significantly to cardiovascular health [2, 3]. Prior work found that patients with CVD have high mortality rates when diagnosed with COVID-19 [14]. The use of e-cigarettes (vaping), or consumption of tobacco has also been shown to increase the risk of infection and progression of COVID-19 [12, 15, 16]. Further, recent studies show that people experiencing substance use disorders (SUD) are both more likely to develop COVID-19 disease as well as experience worse COVID-19 outcomes [17]. Prior work also demonstrated a decrease in physical activity levels in Dutch CVD patients during the first wave of COVID-19 pandemic with an increase in sedentary time [2].

#### 1.1.2 Reddit and health behaviors

Reddit is a publicly available online platform with mostly anonymous and detailed narratives and experiences from over 52 million daily active users participating in more than 100,000 communities (a.k.a. subreddits) [18]. Reddit posts include detailed accounts of individuals’ beliefs, concerns, and behaviors, due to the platform affordances such as user anonymity and freedom of speech [19]. The diverse, profound, and outspoken nature of the discussions on Reddit, make it an apt resource for analyzing individuals’ health behaviors. In the past two years, there have been several research studies assessing different health behaviors using subreddits. Prior data supports that people share their experiences on social media to emotionally cope with pandemic-related anxieties [20–22]. Study on propagation of situational information on social media during COVID-19 with the help of natural language processing has proven to be relevant [23]. Further, changes in people’s emotions and thought patterns during the pandemic were studied to uncover large psychological shifts reflecting three distinct phases of the COVID-19 pandemic [24]. Reddit was also used to characterize and predict shifts from a discussion of mental health to the expression of suicidal ideation [20, 25], albeit pre-pandemic. A study showed that people widely used social media to express their moods [26]. The same study predicted depressed users as well as estimated their depression intensity using computational linguistics [26]. In [27] authors compared 1.53 billion geotagged English tweets with Gallup-Sharecare Well-Being Index survey through 1.73 million phone surveys and concluded that data-driven language models leveraging supervised machine methods generated valid geographical estimates of well-being. County level tweets within the US were used to correlate stressful emotions with health behaviors and socioeconomic characteristics [28]. 15.4 million Facebook messages were assessed to demonstrate that open-vocabulary analyses could yield correlations between personality and behavior as manifested through natural language [29].

### 1.2 Research questions

This paper addresses the following questions:

- How did the engagement, measured by the number of posts and new users, across subreddits associated with diet, physical activity, substance use, and smoking change during 2019 and 2020 (termed as pre- and during pandemic)?
- What discussions, represented using probabilistic generative topic models, were prominent across subreddits associated with diet, physical activity, substance use, and smoking pre-pandemic versus during pandemic?

## 2. Methods

### 2.1 Data

We identified 22 online communities, termed as subreddits on Reddit, that belong to four broader groups—diet, physical activity, substance use, and smoking—and studied how language associated with the behavioral, social, and environmental determinants of physical health changed across 2019 and 2020. For example, subreddits such as r/Drugs, r/addiction, r/1P_LSD, r/alcoholism, r/stopdrinking facilitate discussions on consumption or effects of illicit substances or alcohol or experiences of people upon attempts of quitting marijuana. Similarly, r/bicycling, r/skiing, r/backpacking have discussions about physical activities. Table 1 shows the number of subreddits, posts, and percentage distribution of posts across the four broader groups.

**Table 1:** Data summary of subreddits across broader groups.

Broader Group	Subreddits	Distribution of subreddits	Total number of posts	Distribution of posts	Total number of unique users per broader group
Diet	6	27%	263,647	27%	142,738
Substance Use	7	32%	450,315	46%	193,991
Physical Activity	5	23%	166,812	17%	82,940
Smoking	4	18%	96,850	10%	49,524
Total	22	100%	977,624	100%	469,193

We obtained the data using the Pushshift API [30]. The posts from respective subreddits were scraped using PRAW and PSAW python packages from January 6, 2019, to January 5, 2021. Of the subreddits where individuals were discussing diet, physical activities, substance use, alcohol, or smoking, we identified 22 that had been created before January 2019 and had more than 1,000 posts per subreddit. In total, we obtained 977,624 posts by 435, 041 users in our dataset. WHO published the first Disease Outbreak News on the novel Coronavirus [31] on January 5, 2020, and on January 15, 2020, CDC revealed the first COVID-19 infection in the United States of America [32]. We define the ‘pre-covid’ timeline from January 6, 2019, to January 5, 2020, and the ‘during covid’ timeline from January 6, 2020, to January 5, 2021. The final list of subreddits that were used for the analysis has been mentioned in S1 Table in S1 File.

This analysis was on fully public data and our prior correspondence with University of Pennsylvania IRB verified that such studies did not need approval.

### 2.2 Language feature extraction

Using the DLATK Python package [33], we extracted words and phrases from the entire set of 977,624 posts. Features that were not present in at least 5% of the posts were excluded as outliers. Pointwise mutual information (PMI) [34] was used to retain high occurrence probability features with a threshold of greater than 6. We removed common English stop words from the MALLET package along with most frequent words mentioning COVID-19 and Coronavirus.

Using the MALLET implementation of Latent Dirichlet Allocation (LDA), a set of 50, 100, and 200 topics were created. Coherence scores for each of the three sets of topics were calculated to assess the quality of topics as shown in S13 Table in S1 File. Three coherence measures C_V (0, 1), C_UCI (0,1), and C_NPMI (0,1) were used for evaluation [35]. This was followed by authors’ interpretation of the topics from the top words within each of the three sets. 200 topics were chosen. The probability distribution for each post across all 200 topics was obtained. Each post is represented as a 200-dimensional vector and then aggregated to the level of {user, subreddit, timeframe}, where the time frame is defined as pre- or during COVID-19. The top 15 words and 10 posts most associated with each significant topic were identified by the authors to ascertain the subject matter of the latent topics [36].

### 2.3 Engagement across broader groups

Participation levels within a broader group were measured using the volume of posts made every week [37]. The number of posts were aggregated per week within all subreddits belonging to respective broader groups to represent a broader group’s weekly participation activity level. The influx of new users was assessed by following the methodology from prior work [37], calculated every week within all subreddits belonging to respective broader groups. In order to eliminate the possibility of a general trend (downward/upward) in participation levels and influx of new users and not specifically due to the quarantine we applied Interrupted Time Series (ITS) [37, 38] method on the weekly count of posts and new users to assess the salience of the week of March 23, 2020. In this method we separately regressed the weekly message counts (*y*_*i*_*)* and new user counts (*y*_*i*_*)* data on weeks (*x*_*i*_*)* while also including an indicator variable (1{*x*_*i*_ > *c*}) referred to as ‘pandemic’ response variable henceforth, to indicate the period before the week of March 23, 2020 (coded 0) or the period after the week of March 23, 2020 (coded 1) [39]. *c* indicates the threshold week of March 23, 2020 in the following equation:

$$y_{i}=\beta_{0}+\;\beta_{1}x_{i}+\beta_{2}1\{x_{i}>c\}+\beta_{3}1\{x_{i}>c\}x_{i}$$

This method prevents misinterpretation of a steady increase (or decrease) in time series trend as a treatment effect [38]. Centering the weeks at week of March 23, 2020, was essential. The coefficient (*β*_2_) of the pandemic response variable (indicator variable) after performing the regression helped in quantifying the shift in engagement. We also define a metric to measure the impact percentage (I) of the pandemic response variable (indicator variable) as follows:

$$I=\left(\frac{\beta_{2}+\beta_{0}}{\beta_{0}-1}\right)*100$$

*I* > 0 implies that after the week of March 23, 2020, the engagements (weekly message counts and new user counts) increased whereas *I* < 0 implies otherwise. Larger the magnitude of *I*, more prominent was the impact of pandemic restrictions on the engagement.

### 2.4 Change in language markers pre- and during COVID-19

Language features (words, phrases, and topics) are used as the explanatory variables (features) in ordinary least squares regression, and within each broader group (diet, physical activity, substance use, and smoking) and the time period—pre-pandemic or during the pandemic, which is an indicator variable is used as the pandemic indicator variable. The coefficient of the target explanatory variable is taken as the strength of the relationship. Two-tailed significance values are computed for each coefficient and a Benjamini-Hochberg method of FDR correction is applied [40] to identify significant features (p < 0.001). We used “covid”, “quarantine”, “lockdown”, “coronavirus”, “covid-19”, “pandemic” and “corona” as stop words in the analyses as they were prevalent in most posts during the pandemic.

### 2.5 Temporal trends in topics across broader health behaviors

To identify topics associated with the four broader groups, we repeat the analyses, with features aggregated at the user-subreddit level, but with indicator variables for each of the broader topics as the pandemic indicator variable. We find the Pearson correlations (denoted as ‘r’ henceforth) between topics and the four broader groups (diet, physical activity, substance use, and smoking). For diet, physical activity, substance use, and smoking we found a total of 59, 56, 110, and 53 significant topics respectively, which can be used as the language markers of each broader group. We then analyze the topic distribution over time (2019–2020) for all positively significant topics across the four broader groups.

## 3. Results

To assess the change in engagement across each broader group, we measure participation levels within a broader group by calculating the number of posts made in each week separately for the pre-covid and during COVID-19 time frames. Then, we study changes in words, phrases, and topic features pre- and during COVID-19 and trends in language markers across 2019 and 2020.

### 3.1 Engagement across broader groups

**Fig 1:**
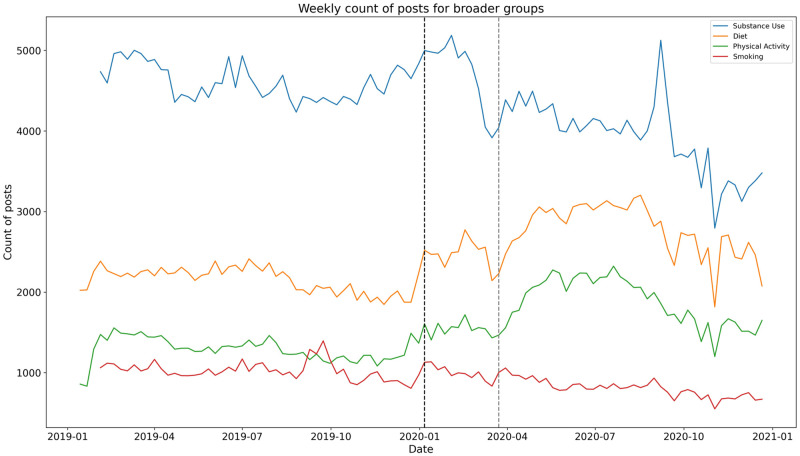
Weekly count of posts across subreddits for four broader groups. The black-colored dashed line signifies the split of pre-pandemic and during the pandemic. The week of March 23, 2020, is highlighted by the grey dashed vertical line.

**Fig 2:**
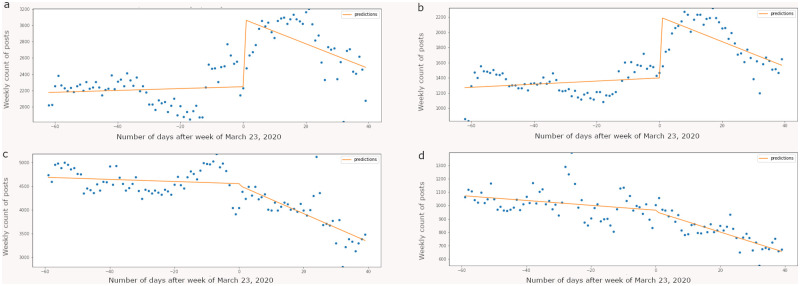
Interrupted time series analysis performed on weekly posts belonging to diet (a), physical activity (b), substance use (c), and smoking (d) broader groups. Y-axis indicates number of posts aggregated at week level across subreddits belonging to their respective broader groups. The discontinuity test was performed on the week of March 23, 2020. For diet and physical activity broader groups, we observe a significant jump (p < 0.001) in the number of weekly posts after the week of March 23, 2020.

Fig 1 represents weekly message counts from the diet, physical activity, substance use, and smoking broader groups. For diet and physical activity broader groups, we observe a significant jump (p < 0.001) in the number of weekly posts after the week of March 23, 2020. It is that week in the month of March, several stay-at-home orders were issued. S11 Table in S1 File exhibits the summary of the ITS regression output suggesting that there is very strong evidence of an increase in weekly message counts for diet (I = 36.90%; p < 0.001) and physical activity (I = 57.70%; p < 0.001) subreddits. For substance use and smoking subreddits the shift in weekly message counts is not significant as also illustrated in Fig 2. Of note, [0.025 and 0.975] are both measurements of values of the coefficients within 95% of our data, or within two standard deviations. In other words, .025 and .975 are 95% confidence intervals of the effect sizes.

**Fig 3:**
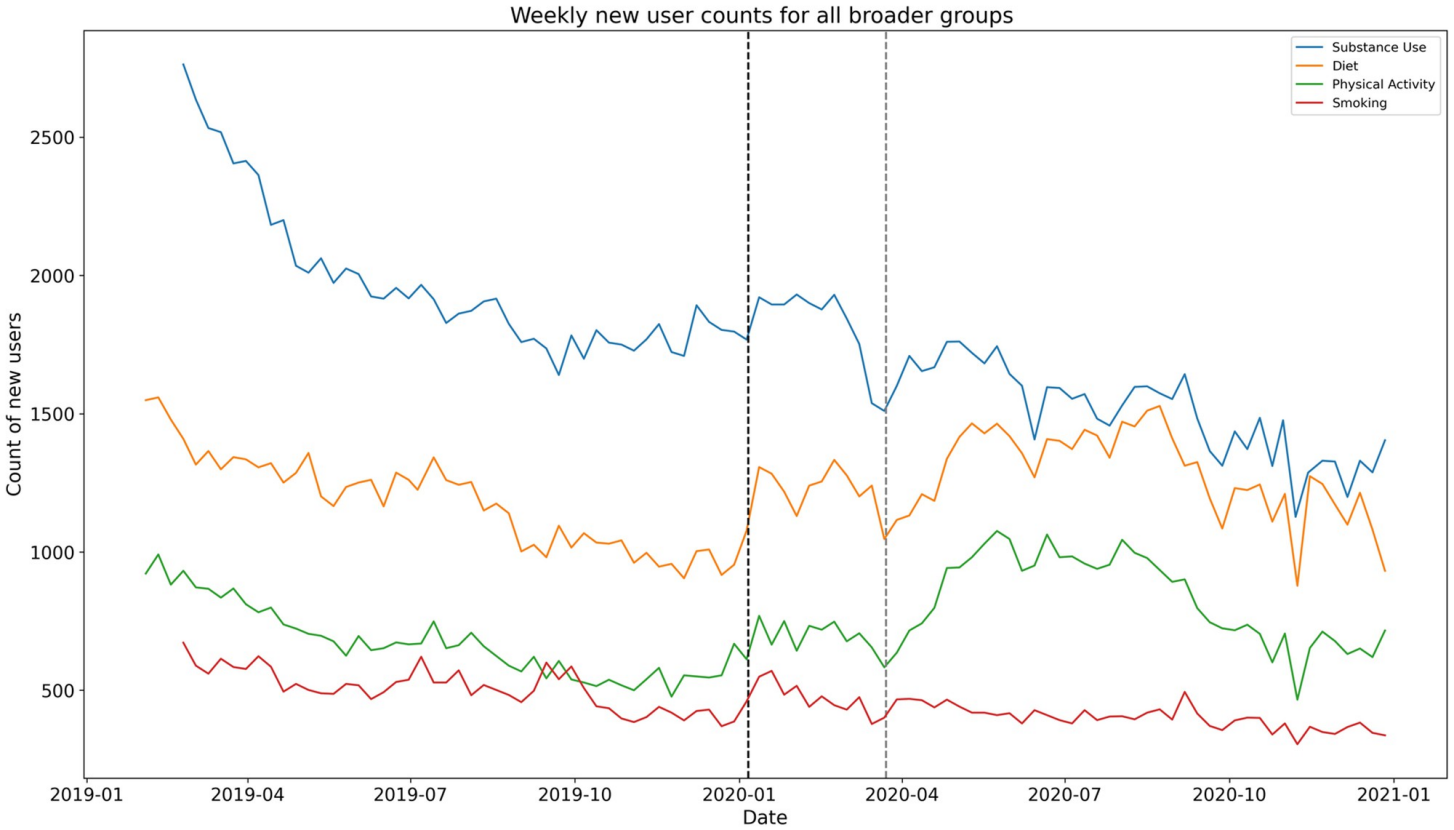
Weekly count of new users across subreddits for four broader groups. The black-colored dashed line signifies the split of pre-pandemic and during the pandemic. The week of March 23, 2020, is highlighted by the grey dashed vertical line.

**Fig 4:**
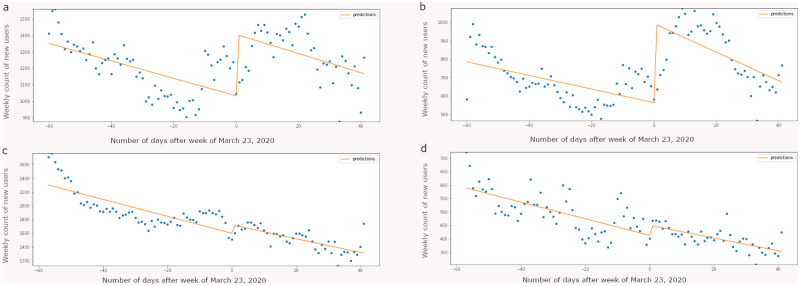
Interrupted time series analysis performed on weekly new user counts belonging to diet (a), physical activity (b), substance use (c), and smoking (d) broader groups. Y-axis indicates number of new users aggregated at week level across subreddits belonging to their respective broader groups. The discontinuity test was performed on the week of March 23, 2020. For diet and physical activity broader groups, we observe a significant jump (p < 0.001) in the number of weekly posts after the week of March 23, 2020.

Fig 3 shows a significant rise in the number of new users (p < 0.001) the week of March 23, 2020, for diet and physical activity subreddits which aligns with the rising weekly message count for each of the two broader groups. S12 Table in S1 File exhibits the summary of the ITS regression output suggesting that there is very strong evidence of an increase in number of new users for diet (I = 36.06%; p < 0.001) and physical activity (I = 76.08%; p < 0.001) subreddits. For substance use and smoking subreddits the shift in weekly message counts is not significant as also illustrated in Fig 4. Of note, [0.025 and 0.975] are both measurements of values of our coefficients within 95% of our data, or within two standard deviations. In other words, .025 and .975 are 95% confidence intervals of the effect sizes.

### 3.2 Change in language markers pre- and during COVID-19

**Fig 5:**
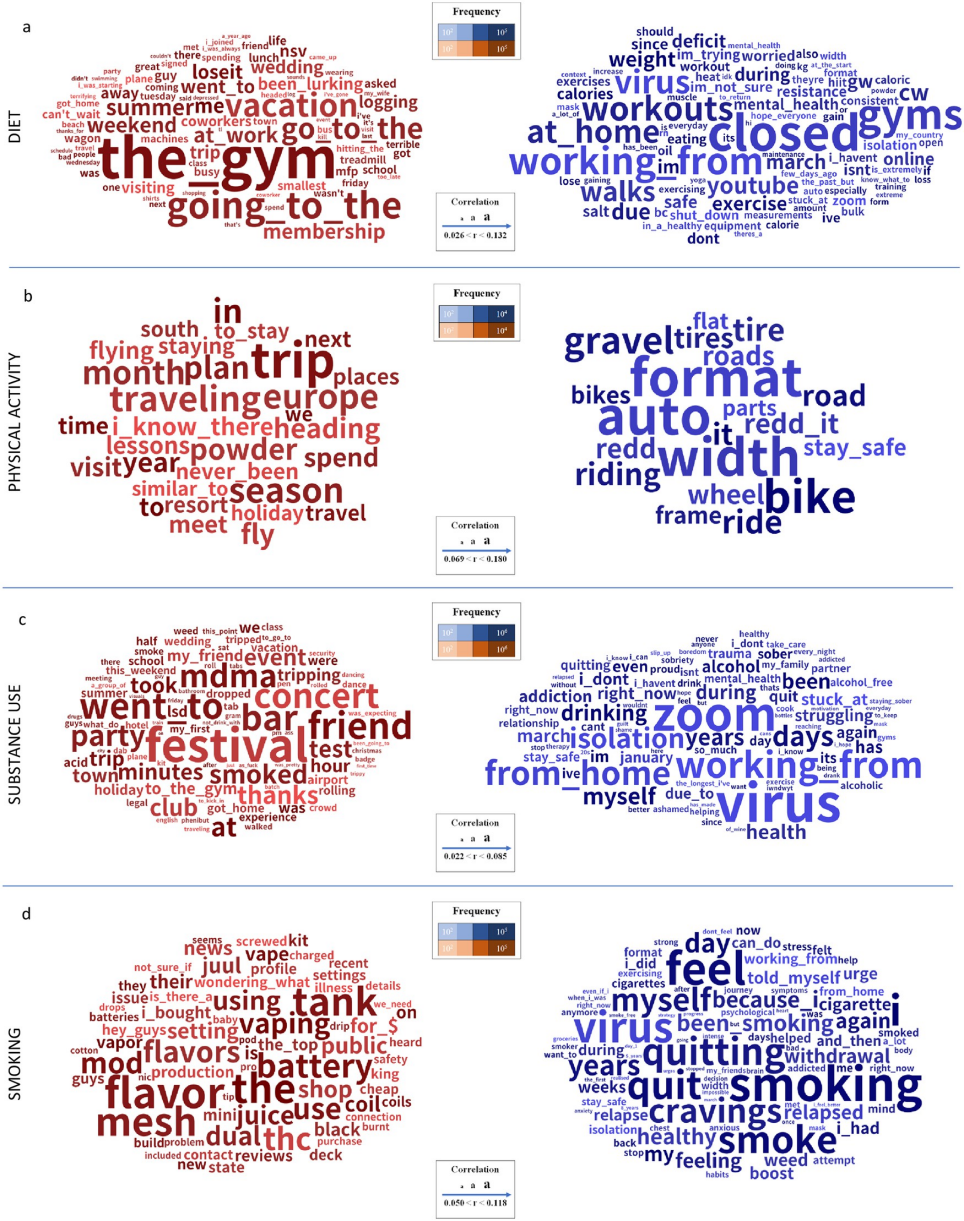
Words and phrases (1to3 grams) significantly associated with diet (a), physical activity (b), substance use (c), and smoking (d) broader groups, most correlated with pre-pandemic (red) and during pandemic (blue), (Benjamini-Hochberg-corrected p < 0.001). Size of the words exhibits magnitude of the correlation value.

**Fig 6:**
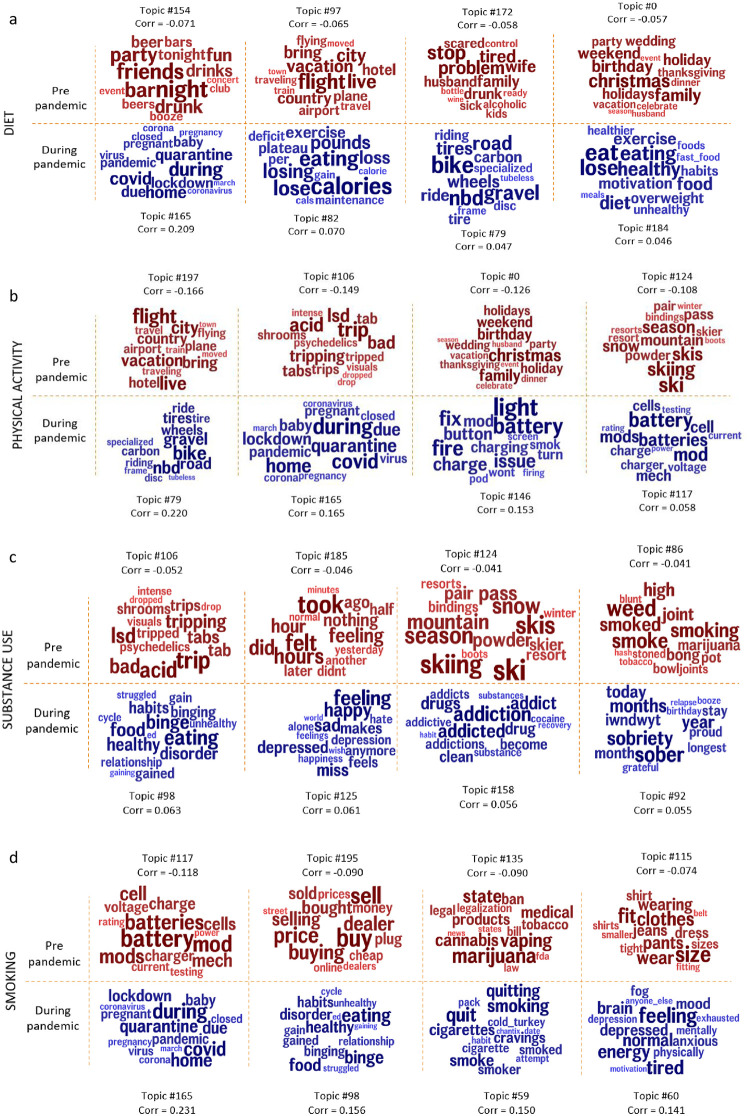
Topics significantly associated with diet (a), physical activity (b), substance use (c), and smoking (d) broader groups, most correlated with pre-pandemic (red) and during pandemic (blue), (Benjamini-Hochberg-corrected p < 0.001). Size of the words exhibits magnitude of the correlation value.

Figs 5 and 6 show words and phrases and topics significantly associated with diet, physical activity, substance use, and smoking broader groups respectively before and during the pandemic. A complete list of the top 15 words for every topic along with associated p-values, effect sizes with the indicator variable, and 95% confidence intervals are provided in S2 Table in S1 File.

Diet: Words and phrases such as ‘going to’, `the gym’, ‘vacation’, ‘summer’, and ‘wedding’ and topics reveal that social drinking (‘night’, ‘friends’, ‘bar’, ‘party’, ‘drunk’, r = -0.071), international travel (‘flight’, ‘live’, ‘city’, ‘vacation’, ‘bring’, r = -0.065), problems due to alcoholism (‘stop’, ‘problem’, ‘tired’, ‘wife’, ‘family’, r = -0.058), family get-together (‘christmas’, ‘family’, ‘birthday’, ‘weekend’, ‘holiday’, r = -0.057) were predominant in pre-pandemic posts. Whereas during the pandemic, ‘at home’ ‘working from’, ‘workouts’, ‘gyms’, ‘closed’, and ‘walks’ and topics about home quarantine (‘during’, ‘covid’, ‘home’, ‘quarantine’, ‘due’, r = 0.209), weight loss (‘calories’, ‘eating’, ‘lose’, ‘losing’, ‘pounds’, r = 0.070), bikes (‘bike’, ‘nbd’, ‘gravel’, ‘road’, ‘tires’, ‘wheels’, r = 0.047), and healthy diet (‘eat’, ‘lose’, ‘eating’, ‘healthy’, ‘diet’, r = 0.046) were predominant.

Physical activity: Words and phrases around physical activity were ‘trip’, ‘traveling’, ‘season’, ‘europe’, ‘month’, ‘flying’, and ‘holiday’ and topics about international travel (‘flight’, ‘live’, ‘city’, ‘vacation’, ‘bring’, r = -0.166), substance use (‘trip’, ‘acid’, ‘lsd’, ‘bad’, ‘tripping’, r = -0.149), family get-together (‘christmas’, ‘family’, ‘birthday’, ‘weekend’, ‘holiday’, r = -0.126), and skiing (‘ski’, ‘skiing’, ‘skis’, ‘season’, ‘snow’, r = -0.108) were dominant pre-pandemic. During the pandemic, words and phrases such as ‘bike’, ‘auto’, ‘format’, ‘riding’, ‘gravel’, and ‘format’ and topics about biking (‘bike’, ‘nbd’, ‘gravel’, ‘road’, ‘tires’, r = 0.220), home quarantine (‘during’, ‘covid’, ‘home’, ‘quarantine’, ‘due’, r = 0.165), electronic vape parts (‘light’, ‘battery’, ‘fire’, ‘fix’, ‘issue’, r = 0.153), and vape batteries (‘battery’, ‘mod’, ‘batteries’, ‘mods’, ‘cell’, r = 0.058) were dominant.

Substance use: Words and phrases in substance use subreddits pre-pandemic were ‘festival’, ‘concert’, ‘mdma’, ‘smoked’, ‘bar’, ‘party’, ‘club’ and topics were about substances (‘trip’, ‘acid’, ‘lsd’, ‘bad’, ‘tripping’, r = -0.052), after-effects of substance use (‘took’, ‘felt’, ‘hours’, ‘did’, ‘feeling’, r = -0.046), skiing (‘ski’, ‘skiing’, ‘skis’, ‘season’, ‘snow’, r = -0.041), and marijuana (‘weed’, ‘smoke’, ‘smoking’, ‘smoked’, ‘high’, r = -0.041). During the pandemic, words and phrases such as ‘virus’, ‘zoom’, ‘isolation’, ‘working from’, ‘from home’, ‘days’, ‘myself’ and topics about binge eating (‘eating’, ‘binge’, ‘food’, ‘healthy’, ‘disorder’, r = 0.63), feeling emotional (‘feeling’, ‘happy’, ‘sad’, ‘miss’, ‘depressed’, r = 0.061), addiction (‘addiction’, ‘addicted’, ‘addict’, ‘drugs’, ‘drug’, r = 0.056), and staying sober (‘sober’, ‘sobriety’, ‘months’, ‘year’, ‘today’, r = 0.055) were predominant.

Smoking: Words and phrases in the smoking subreddits such as ‘flavor’, ‘mesh’, ‘tank’, ‘battery’, ‘vaping’, ‘mod’, ‘coil’ and topics about vape batteries (‘battery’, ‘mod’, ‘batteries’, ‘mods’, ’cell’, r = -0.118), transacting (‘buy’, ‘sell’, ‘price’, ‘buying’, ‘dealer’, r = -0.090), marijuana (‘marijuana’, ‘vaping’, ‘state’, ‘cannabis’, ‘medical’, r = -0.090), and physique and clothing size (‘size’, ‘fit’, ‘clothes’, ‘wear’, ‘pants’, r = -0.074) were dominant pre-pandemic. During the pandemic, words and phrases such as ‘smoking’, ‘feel’, ‘virus’, ‘quitting’, ‘cravings’ and topics such as home quarantine (‘during’, ‘covid’, ‘home’, ‘quarantine’, ‘due’, r = 0.231), binge eating (‘eating’, ‘binge’, ‘food’, ‘healthy’, ‘disorder’, r = 0.156), quitting smoking (‘quit’, ‘smoking’, ‘quitting’, ‘smoke’, ‘cigarettes’, r = 0.150), and mental exhaustion (‘feeling’, ‘tired’, ‘energy’, ‘normal’, ‘brain’, r = 0.141) were dominant.

### 3.3 Temporal trends in topics across broader health behaviors

**Fig 7:**
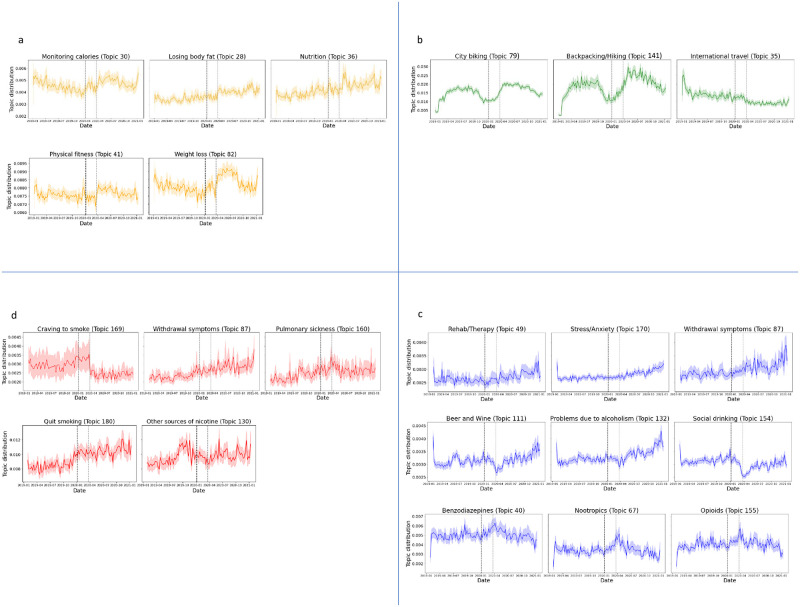
Prevalent topics based on posts belonging to diet (a), physical activity (b), substance use (c), and smoking (d) broader groups. Y-axis values indicate the probability that any post within the broader group at a given week contains the corresponding topic. Values have been reported as a 7-day rolling average for each week. The shaded region corresponds to the 95% confidence interval. The grey vertical dashed line signifies the week of March 23, 2020. Black vertical dashed line demarcates pre-pandemic and during pandemic datasets.

Fig 7 depicts the trends in prevalent (significant) topics across four broader groups, diet, physical activity, substance use, and smoking.

Diet: Topics on weight loss (‘calories’, ‘eating’, ‘lose’, ‘losing’, ‘pounds’, r = 0.070), fitness (‘pounds’, ‘lost’, ‘gained’, ‘healthy’, ‘overweight’, r = 0.031), nutrition (‘diet’, ‘protein’, ‘carbs’, ‘sugar’, ‘fat’, r = 0.031), calorie monitoring (‘intake’, ‘bmr’, ‘caloric’, ‘count’, ‘maintenance’, r = 0.046), and cutting body fat (‘lose’, ‘fat’, ‘body’, ‘diet’, ‘metabolism’, r = 0.059) were observed to increase after the week of March 23, 2020.

Physical activity: Discussions within physical activity subreddits exhibit seasonality in quite a few topics. Biking (‘bike’, ‘ride’, ‘road’, ‘riding’, ‘cycling’, r = 0.22), and hiking (‘mountain’, ‘miles’, hike, ‘backpack’, ‘trail’, r = 0.175) observe an increase after the week of March 23, 2020, followed by a gradual decline till December 2020. International travel (‘trip’, ‘travel’, ‘asia’, ‘countries’, ‘thailand’, r = -0.162) maintains a decreasing trend after the week of March 23, 2020.

Substance use: Discussions of therapy (‘rehab’, ‘detox’, ‘treatment’, ‘hospital’, ‘therapy’, r = 0.033), stress (‘stress’, ‘anxiety’, ‘deal’, ‘feelings’, ‘emotions’, r = 0.065), withdrawal symptoms (‘symptoms’, ‘withdrawals’, ‘cold turkey’, ‘shakes’, ‘seizures’, r = 0.026), beer/wine (‘beer’, ‘wine’, ‘bottle’, ‘iwndwyt’, ‘glass of wine’, r = 0.029), problems related to alcoholism (‘problem’, ‘alcoholic’, ‘drunk’, ‘drank’, ‘alcoholism’, r = 0.062), and social drinking (‘night’, ‘friends’, ‘bar’, ‘party’, ‘drunk’, r = -0.056) increase in prevalence after the week of March 23, 2020 and topics about substances such as benzodiazepines (‘xanax’, ‘took’, ‘benzos’, ‘valium’, ‘mg’, r = -0.018), nootropics (‘dxm’, ‘kratom’, ‘phenibut’, ‘dph’, ‘benadryl’, r = -0.033), and opioids (‘oxycodone’, ‘opiates’, ‘pill’, ‘morphine’, ‘opioids’, r = -0.006) increase from the week of January 6, 2020, until the week of March 23, 2020, and decline gradually over the next four weeks.

Smoking: Topics about withdrawal symptoms (‘symptoms’, ‘withdrawals’, ‘cold turkey’, ‘shakes’, ‘seizures’, r = 0.125) and attempts to quit smoking (‘quit’, ‘cigarette’, ‘pack’, ‘quitting’, ‘cravings’, r = 0.140) exhibit a rising trend whereas discussions on pulmonary sickness (‘throat’, ‘sick’, ‘chest’, ‘lungs’, ‘cough’, ‘breathing’, r = 0.097) drop over 8–10 weeks after the week of March 23, 2020. Craving to smoke (‘craving’, ‘strong’, ‘urge’, ‘thoughts’, ‘fight’, r = 0.130) topic witnesses a steep drop whereas users rebuild their interest in discussions about other sources of nicotine (‘nicotine’, ‘juul’, ‘tobacco’, ‘gum’, ‘patch’, r = 0.026) after the week of March 23, 2020.

## 4. Discussion

Analyses of Reddit posts revealed significant changes in health behavior expressions around diet, physical activity, substance use, and smoking before and during the COVID-19 pandemic. The changes were reflected in both engagement, measured by the number of posts and influx of new users, and content of posts, measured by words and phrases and thematic content.

In the week of March 23, 2020, several stay-at-home orders were issued [41]. While there was heterogeneity in the way policies were enacted in each country and state, around this week, a paradigm shift in social and physical behavior had been initiated. The lockdown mandate implied restaurants, gyms, sports complexes, had to shut their doors to in-person customers to prevent the spread of COVID-19. This transition also was mirrored in discussions online, both in terms of engagement and participation levels across the 22 subreddits associated with diet, physical activity, substance use, and smoking, and the thematic content of users’ posts within those categories.

The first research question in this paper assessed how engagement levels within each broader group changed after the COVID-19 pandemic outbreak. We observed a significant increase in the volume of weekly posts made after the week of March 23, 2020, across the diet and physical activity subreddit categories. The impact percentage suggests that the week of March 23, 2020, led to 36.90% and 57.70% increase in weekly posts within diet and physical activity subreddits. This increase could potentially be due to either existing users posting about their diet and physical activity at a much higher rate during the COVID-19 pandemic or new users joining the diet and physical activity subreddits contributing to the surge in activity levels. For each broader category, the factor underlying the change in engagement levels from pre- to during pandemic became clear by analyzing the weekly new user influx from 2019 to 2021. We observed a significant increase in the number of new users after the week of March 23, 2020, for the diet and physical activity subreddits aligning with the upsurge in weekly activity for each of the two broader groups. The impact percentage suggests that the week of March 23, 2020, led to 36.06% and 76.08% increase in weekly new users within diet and physical activity subreddits. This suggests that the proportion of users interested in diet and exercise may have broadened.

There were notable thematic differences in content between subreddits within each category before and during the COVID-19 pandemic. Upon observing the thematic topics, we found language markers ranging from generic topics corresponding to themes such as vacation/travel, work, family, to specific topics corresponding to themes such as substance use, bike parts, vape parts, withdrawal symptoms, and pulmonary sickness. We found factual topics exhibiting general information such as the ban on illicit substances by the government, vape parts, purchase of illicit substances, bike parts, etc. and emotionally charged topics about attempts to quit smoking, withdrawal symptoms, binge eating, anxiety, and craving to smoke. The analysis revealed that users were posting more about emotions and feelings during the pandemic versus before the pandemic they focused more on facts and details.

Temporal trends of significant prevalent topics revealed that Reddit users after the week of March 23, 2020, discussed more about nutrition in diet, physical fitness, and outdoor activities such as backpacking and biking. This suggests that the pandemic and social isolation could have potentially driven users to place increased focus on their physical health. After observing prevalent topic distributions from substance use subreddits we can infer that users wrote more about coping with stress and anxiety, alcohol misuse and how it was affecting their lives, and switching to beer and wine from other forms of alcohol after the isolation began from the week of March 23, 2020. Exposure to stress in different forms is closely associated with subsequent alcohol consumption. In fact, individuals with a history of alcohol use disorder (AUD) are more likely to drink to cope with different traumatic events [42].

A study on substance use during the pandemic cites a reporting system, ODMAP, that shows an 18% increase in substance overdoses in the US in the early months of the pandemic compared with those same months in 2019 [43]. Our results showed a similar spike in the discussions on substances such as benzodiazepines (valium, xanax, clonazepam), nootropics (kratom, phenibut), and opioids (fentanyl, oxycodone, morphine) around the week of March 23, 2020. Illicit drug unavailability and increased mental stress during the isolation may have driven the discussions toward the consumption of opioid alternatives such as fentanyl, kratom, etc., which we observed. However, increase in discussions after the week of March 23, 2020, about rehabilitation, therapy, and withdrawal symptoms hint at users attempting to quit alcohol and illicit substances.

Prevalent topics from smoking subreddits showed that the onset of pandemic led to increase in discussions around quitting smoking, analyzing other sources of nicotine such as nicotine gums and patches, and withdrawal symptoms. Further, discussions on their cravings to smoke saw a decline after the week of March 23, 2020. All these observations possibly hint that users were aware of pulmonary risks associated with COVID-19 and sought attempts to quit smoking or switch to other forms of nicotine.

### 4.1 Limitations

A recent study [42] reported a 54% increase in national sales of alcohol for the week ending March 21, 2020, compared with 1 year before in the United States. Online alcohol sales had experienced a surge of 262% from 2019 in the US. The Federal Trade Commission reported a 0.4% increase in cigarette sales in the US from 202.9 billion in 2019 to 203.7 billion in 2020 [44]. The increase in tobacco sales had been observed for the first time in two decades. The unavailability of geographic locations in Reddit limited our study from analyzing changes in language markers from posts at the state or county level and comparing those with offline statistics such as cardiovascular health outcomes. Like most social media studies, our data is not representative of the general population even though Reddit is one of the most popular platforms in the US. In [45] we find that approximately 50% of the Reddit traffic flows from the US. Reddit being an anonymous forum does not reveal demographics of users and we extrapolate the country traffic distribution on our dataset. While we included most subreddits that deal with the four broader health behaviors of interest to this study with reasonable participation, we could have missed some. We would like to further highlight that the focus of the research was not on the intricacies of the nature of the four health behaviors while shortlisting the subreddits. As observed in S1 Table in S1 File, Physical Activity broader group may have been biased with outdoor physical activity subreddits compared to indoor physical activity subreddits such as Yoga or HIIT. Similarly, certain substances might be easier to obtain and might have contributed more to the discussions within subreddits from Substance Use broader group. Moreover, similar to self-reporting on health behaviors, posting about quitting smoking, illicit substances, or alcohol is not evidence that the users have quit smoking, stopped alcohol consumption, or ceased substance use. This conversation-action gap could potentially be alleviated by considering multiple modalities of user data, while it potentially comes at the cost of user privacy. Social media conversations are a complementary source to the data obtained by traditional surveys and further provide insights that could potentially not be obtained from traditional methods owing to their paradigm.

Public health events such as COVID-19 that resulted in isolation and quarantine had a substantial impact on population physical and mental health globally. Our study highlights the importance of social media discussions on platforms like Reddit that offer ecological and large-scale data to summarize and potentially measure changes in health behaviors. The candid and open expressions on social media data could well complement the rich traditional data collection methods and further provide insights for real-time public health infoveillance, and targeted messaging at the times of crises.

Pre-pandemic and during pandemic language comparisons to suggest changes in health behaviors in this paper are made over several weeks and many sociopolitical factors over time could have influenced results in different ways. For example, changes in tax rates of cigarettes or alcohol or impact of protests and elections in the US during the timeframe of our study. Such situations might have impacted the size of and direction of language markers over time. We do not assume that COVID-19 pandemic is the sole cause of the changes in health behaviors depicted through social media language, but rather that these changes are only associated to the pandemic. Of note, there are methods from causal inference for observational studies such as propensity score matching that could be leveraged to go beyond association claims and strengthen causal claims.

Topic models are algorithms that aid in discovering important themes from a large and unstructured collection of documents [46]. Latent Dirichlet Allocation is one of the simplest topic modelling algorithms that belongs to the class of generative probabilistic models [46]. It however has its fair share of limitations. Topic modeling has evolved over time and newer more sophisticated techniques such as [47, 48] for topic modeling leveraging deep learning embeddings are available.

## Supporting information

S1 File Contains supplementary tables. (DOCX)
