# Supplementary material for: Reddit language indicates changes associated with diet, physical activity, substance use, and smoking during COVID-19
Source: PLoS One. 2023 Feb 3;18(2):e0280337. doi: 10.1371/journal.pone.0280337 (PMC9897548; doi:10.1371/journal.pone.0280337)
Supplement: S1 File — (DOCX) [file pone.0280337.s001.docx]

Supplementary Table S1: List of subreddits and their respective broader group

| Substance Use | Physical Activity | Diet | Smoking |
| --- | --- | --- | --- |
| 1P_LSD | trailrunning | diet | stopsmoking |
| Drugs | gravelcycling | EatCheap-AndHealthy | quittingsmoking |
| Marijuana | bicycling | progresspics | smokingcessation |
| addiction | skiing | loseit | electronic_cigarette |
| DrugCombos | backpacking | GettingShredded |  |
| stopdrinking |  | GYM |  |
| alcoholism |  |  |  |

Supplementary Table S2: Topic IDs, top 15 words, and respective correlation with ‘during pandemic’ period. Positive correlation value implies the topic correlates with during pandemic posts, and negative correlation value implies the topic correlates with pre-pandemic posts

|  |  | **Diet** | | | | **Substance Use** | | | | **Physical Activity** | | | | **Smoking** | | | |
| --- | --- | --- | --- | --- | --- | --- | --- | --- | --- | --- | --- | --- | --- | --- | --- | --- | --- |
| **Topic ID** | **Top Words** | **Correlation with during pandemic period** | **p** | **CI_l** | **CI_u** | **Correlation with during pandemic period** | **p** | **CI_l** | **CI_u** | **Correlation with during pandemic period** | **p** | **CI_l** | **CI_u** | **Correlation with during pandemic period** | **p** | **CI_l** | **CI_u** |
| 0 | christmas, family, birthday, weekend, holiday, holidays, wedding, party, thanksgiving, vacation, dinner, celebrate, husband, event, season, gift | -0.057 | <0.001 | -0.072 | -0.041 | -0.016 | 0.003 | -0.026 | -0.006 | -0.126 | <0.001 | -0.162 | -0.089 | 0.013 | 0.434 | -0.017 | 0.043 |
| 1 | she, her, mom, shes, she's, sister, mother, herself, says, wants, girlfriend, mum, knows, thinks, daughter, tells | -0.014 | 0.13 | -0.029 | 0.002 | 0.013 | 0.017 | 0.003 | 0.023 | -0.018 | 0.491 | -0.055 | 0.019 | 0.023 | 0.166 | -0.007 | 0.052 |
| 2 | started, since, age, old, 18, 16, 15, turned, parents, school, 14, 17, stopped, 21, became, everyday | -0.013 | 0.16 | -0.028 | 0.003 | 0.026 | <0.001 | 0.016 | 0.036 | -0.099 | <0.001 | -0.135 | -0.062 | 0.112 | <0.001 | 0.082 | 0.141 |
| 3 | less, energy, loseit, perfect, trick, important, create, term, needs, index, asked, store, meal, pizza, create a deficit, your calorie needs | -0.003 | 0.73 | -0.019 | 0.012 | 0.029 | <0.001 | 0.019 | 0.039 | 0.002 | 0.960 | -0.035 | 0.039 | -0.002 | 0.904 | -0.032 | 0.027 |
| 4 | than, better, less, lot, easier, harder, longer, worse, feels, definitely, noticed, stronger, expected, gotten, difference, changed | -0.011 | 0.23 | -0.026 | 0.005 | 0.040 | <0.001 | 0.030 | 0.050 | -0.042 | 0.062 | -0.079 | -0.005 | 0.075 | <0.001 | 0.046 | 0.105 |
| 5 | stopdrinking, give, daily, pledge, you're, simple, we're, next 24 hours, happens, lets, ready, decide, show, posting, decision, internet | -0.021 | 0.01 | -0.037 | -0.006 | 0.013 | 0.018 | 0.003 | 0.023 | -0.017 | 0.506 | -0.054 | 0.020 | 0.020 | 0.233 | -0.010 | 0.049 |
| 6 | our, us, were, both, each, together, we're, ourselves, we've, group, weve, husband, our lives, partner, we'd, neither | -0.025 | 0.00 | -0.041 | -0.010 | -0.015 | 0.006 | -0.025 | -0.005 | -0.054 | 0.015 | -0.091 | -0.018 | -0.044 | 0.006 | -0.073 | -0.014 |
| 7 | his, guy, car, fuck, walk, story, walking, man, says, dude, call, starts, door, street, outside, bag | -0.043 | <0.001 | -0.058 | -0.028 | -0.056 | <0.001 | -0.066 | -0.046 | -0.055 | 0.014 | -0.092 | -0.018 | -0.042 | 0.008 | -0.072 | -0.013 |
| 8 | chicken, rice, recipe, beans, meat, recipes, sauce, cheap, soup, veggies, salad, pasta, cheese, cook, potatoes, vegetables | 0.014 | 0.13 | -0.002 | 0.029 | -0.012 | 0.028 | -0.022 | -0.002 | -0.007 | 0.783 | -0.044 | 0.030 | -0.052 | 0.001 | -0.082 | -0.022 |
| 9 | food, healthy, meals, cook, recipes, meal, ideas, cooking, easy, cheap, foods, suggestions, budget, lunch, kitchen, fridge | 0.011 | 0.23 | -0.005 | 0.026 | 0.019 | <0.001 | 0.009 | 0.029 | -0.042 | 0.058 | -0.079 | -0.006 | 0.001 | 0.970 | -0.029 | 0.030 |
| 10 | eat, food, eating, ate, pizza, sugar, chocolate, cake, ice cream, cookies, chips, candy, sweets, snacks, junk food, sweet | 0.007 | 0.48 | -0.009 | 0.022 | 0.001 | 0.927 | -0.010 | 0.011 | 0.034 | 0.137 | -0.003 | 0.071 | 0.025 | 0.122 | -0.004 | 0.055 |
| 11 | car, driving, drive, police, jail, dui, cops, license, pulled, court, arrested, driver, drove, cop, caught, cars | -0.034 | <0.001 | -0.049 | -0.018 | -0.028 | <0.001 | -0.038 | -0.018 | -0.029 | 0.215 | -0.066 | 0.008 | -0.067 | <0.001 | -0.097 | -0.038 |
| 12 | kg, 10, old, 20, 15, kgs, 30, cm, age, 14, 25, 12, 18, 60, 100, 13 | 0.030 | <0.001 | 0.014 | 0.045 | 0.011 | 0.043 | 0.001 | 0.021 | 0.015 | 0.554 | -0.022 | 0.052 | 0.011 | 0.507 | -0.018 | 0.041 |
| 13 | book, read, reading, mind, books, naked, helped, podcast, recommend, listening, podcasts, writing, write, helpful, listen, wrote | -0.014 | 0.12 | -0.029 | 0.002 | 0.018 | 0.001 | 0.008 | 0.028 | -0.050 | 0.027 | -0.087 | -0.013 | 0.027 | 0.104 | -0.003 | 0.056 |
| 14 | sorry, post, english, wrong, sub, reddit, ask, language, hello, mobile, what's, advance, posting, subreddit, rules, write | 0.016 | 0.08 | 0.000 | 0.031 | 0.000 | 1.000 | -0.010 | 0.010 | 0.163 | <0.001 | 0.127 | 0.199 | 0.009 | 0.609 | -0.021 | 0.039 |
| 15 | advice, tips, need, anyone, appreciated, looking, thanks, thank, advance, suggestions, hi, appreciate, hello, tricks, helpful, be greatly appreciated | 0.018 | 0.04 | 0.002 | 0.033 | 0.004 | 0.434 | -0.006 | 0.015 | -0.051 | 0.024 | -0.088 | -0.014 | -0.002 | 0.934 | -0.031 | 0.028 |
| 16 | lbs, months, progress, difference, pic, picture, 150, nsfw, cico, gw, lb, 145, pictures, pics, 175, 180 | 0.013 | 0.16 | -0.003 | 0.028 | 0.028 | <0.001 | 0.018 | 0.038 | 0.018 | 0.486 | -0.019 | 0.055 | 0.056 | <0.001 | 0.026 | 0.086 |
| 17 | week, weekend, friday, saturday, night, sunday, monday, morning, thursday, tuesday, wednesday, weekends, evening, afternoon, nights, sat | -0.053 | <0.001 | -0.068 | -0.037 | -0.022 | <0.001 | -0.032 | -0.012 | -0.059 | 0.008 | -0.096 | -0.022 | 0.002 | 0.903 | -0.027 | 0.032 |
| 18 | post, please, place, interesting, fit, daily, awesomeness, short, reddit, reach, often, set, flair, means, pass, space | -0.019 | 0.03 | -0.035 | -0.004 | 0.021 | <0.001 | 0.011 | 0.032 | 0.006 | 0.836 | -0.031 | 0.043 | 0.027 | 0.104 | -0.003 | 0.056 |
| 19 | trail, hike, backpacking, lake, mountain, hiking, de, mountains, la, mt, trails, view, beautiful, trek, park, camp | -0.002 | 0.85 | -0.018 | 0.013 | -0.032 | <0.001 | -0.042 | -0.022 | -0.012 | 0.634 | -0.049 | 0.025 | -0.062 | <0.001 | -0.091 | -0.032 |
| 20 | again, months, started, year, ago, few, weeks, went, stopped, month, couple, past, decided, break, became, began | -0.019 | 0.03 | -0.034 | -0.003 | 0.025 | <0.001 | 0.015 | 0.035 | -0.078 | <0.001 | -0.115 | -0.041 | 0.114 | <0.001 | 0.085 | 0.143 |
| 21 | doctor, blood, liver, health, heart, blood pressure, doctors, hospital, medical, disease, results, diabetes, levels, damage, diagnosed, er | 0.001 | 0.95 | -0.015 | 0.016 | 0.016 | 0.002 | 0.006 | 0.026 | -0.002 | 0.960 | -0.039 | 0.035 | 0.048 | 0.003 | 0.018 | 0.077 |
| 22 | taking, prescribed, medication, doctor, anxiety, meds, effects, side, prescription, depression, antidepressants, zoloft, ssri, wellbutrin, psychiatrist, dose | 0.010 | 0.28 | -0.006 | 0.025 | -0.006 | 0.312 | -0.016 | 0.004 | -0.043 | 0.057 | -0.080 | -0.006 | 0.041 | 0.010 | 0.011 | 0.071 |
| 23 | water, coffee, tea, caffeine, soda, drinks, sugar, energy, cup, cups, green tea, hot, lemon, milk, apple, ice | 0.011 | 0.21 | -0.004 | 0.027 | -0.005 | 0.419 | -0.015 | 0.005 | 0.004 | 0.873 | -0.033 | 0.041 | 0.002 | 0.929 | -0.028 | 0.031 |
| 24 | change, habits, yourself, habit, important, mental, focus, changes, positive, easier, physical, learn, decision, effort, routine, process | -0.001 | 0.96 | -0.016 | 0.015 | 0.048 | <0.001 | 0.038 | 0.058 | -0.018 | 0.491 | -0.055 | 0.019 | 0.111 | <0.001 | 0.082 | 0.140 |
| 25 | coke, cocaine, ketamine, line, lines, ket, gram, blow, comedown, party, bumps, bump, bag, snorted, grams, nose | -0.007 | 0.49 | -0.022 | 0.009 | -0.032 | <0.001 | -0.042 | -0.022 | -0.024 | 0.307 | -0.061 | 0.013 | 0.027 | 0.105 | -0.003 | 0.056 |
| 26 | months, progress, goal, difference, picture, proud, pic, 165lbs, 200lbs, nsfw, 170lbs, 40lbs, 175lbs, pictures, 220lbs, ' 11 | 0.006 | 0.54 | -0.010 | 0.021 | 0.043 | <0.001 | 0.033 | 0.053 | -0.008 | 0.776 | -0.045 | 0.029 | 0.086 | <0.001 | 0.057 | 0.116 |
| 27 | daily, join, us, yourself, everyone, share, goals, chat, european, you're, wants, welcome, track, wrong, weekly, friendly | -0.026 | 0.00 | -0.042 | -0.011 | 0.019 | <0.001 | 0.009 | 0.029 | -0.045 | 0.047 | -0.082 | -0.008 | 0.052 | 0.001 | 0.022 | 0.081 |
| 28 | loss, lose, fat, body, diet, losing, exercise, gain, fast, burn, energy, metabolism, results, fitness, effective, diets | 0.059 | <0.001 | 0.043 | 0.074 | 0.024 | <0.001 | 0.014 | 0.034 | -0.012 | 0.638 | -0.049 | 0.025 | 0.038 | 0.018 | 0.009 | 0.068 |
| 29 | can't, hate, fucking, shit, stop, anymore, fuck, i'll, sick, won't, literally, tired, die, sorry, worse, wish | -0.036 | <0.001 | -0.052 | -0.021 | 0.037 | <0.001 | 0.027 | 0.047 | -0.030 | 0.192 | -0.067 | 0.007 | 0.080 | <0.001 | 0.050 | 0.109 |
| 30 | calories, per, calorie, tdee, deficit, burn, burned, average, kcal, intake, bmr, caloric, count, maintenance, 1200, calculator | 0.046 | <0.001 | 0.030 | 0.061 | -0.001 | 0.861 | -0.011 | 0.009 | 0.049 | 0.030 | 0.012 | 0.086 | -0.046 | 0.004 | -0.076 | -0.017 |
| 31 | ride, gravel, century, miles, trail, beautiful, riding, cycling, weather, trails, bridge, winter, park, rain, metric, snow | -0.004 | 0.67 | -0.020 | 0.011 | -0.037 | <0.001 | -0.047 | -0.027 | 0.126 | <0.001 | 0.089 | 0.162 | -0.028 | 0.094 | -0.057 | 0.002 |
| 32 | app, phone, apps, track, data, garmin, watch, strava, computer, google, smart, gps, tracking, trainer, map, wahoo | 0.001 | 0.96 | -0.015 | 0.016 | 0.000 | 0.953 | -0.010 | 0.010 | 0.051 | 0.025 | 0.014 | 0.088 | -0.048 | 0.003 | -0.077 | -0.018 |
| 33 | kg, lost, progress, stone, cm, gained, 10kg, 5kg, weigh, 70kg, lockdown, 100kg, 80kg, 90kg, 60kg, kgs | 0.049 | <0.001 | 0.033 | 0.064 | 0.047 | <0.001 | 0.036 | 0.057 | 0.017 | 0.511 | -0.020 | 0.053 | 0.125 | <0.001 | 0.096 | 0.154 |
| 34 | again, yesterday, relapse, drank, wagon, relapsed, reset, fell, badge, slip, disappointed, slipped, streak, broke, messed up, fall | -0.042 | <0.001 | -0.058 | -0.027 | 0.043 | <0.001 | 0.033 | 0.053 | -0.016 | 0.513 | -0.053 | 0.021 | 0.106 | <0.001 | 0.077 | 0.135 |
| 35 | trip, travel, places, backpacking, europe, south, visit, planning, country, traveling, city, asia, countries, india, thailand, travelling | -0.047 | <0.001 | -0.062 | -0.031 | -0.064 | <0.001 | -0.074 | -0.054 | -0.162 | <0.001 | -0.198 | -0.126 | -0.047 | 0.003 | -0.077 | -0.017 |
| 36 | diet, protein, carbs, sugar, fat, foods, low, pd, meat, keto, nutrition, carb, vegan, vegetables, fats, intake | 0.031 | <0.001 | 0.015 | 0.046 | 0.009 | 0.115 | -0.001 | 0.019 | 0.006 | 0.841 | -0.031 | 0.043 | 0.002 | 0.929 | -0.028 | 0.031 |
| 37 | sets, reps, gym, bench, workout, exercises, chest, squat, pull, form, push, leg, squats, deadlift, legs, set | 0.034 | <0.001 | 0.018 | 0.049 | -0.024 | <0.001 | -0.034 | -0.014 | 0.017 | 0.511 | -0.020 | 0.054 | 0.037 | 0.021 | 0.007 | 0.067 |
| 38 | pounds, lost, lbs, lose, 20, 10, 100, 50, 30, 40, 200, gained, 15, goal, 180, 60 | 0.003 | 0.79 | -0.013 | 0.018 | 0.008 | 0.172 | -0.002 | 0.018 | -0.002 | 0.937 | -0.039 | 0.035 | 0.003 | 0.870 | -0.026 | 0.033 |
| 39 | comments, post, loseit, challenge, update, thread, team, posted, activity, step, previous, welcome, posts, note, steps, club | -0.015 | 0.10 | -0.030 | 0.001 | -0.004 | 0.440 | -0.014 | 0.006 | 0.001 | 0.961 | -0.036 | 0.038 | -0.021 | 0.209 | -0.050 | 0.009 |
| 40 | xanax, take, took, benzos, taking, valium, mg, benzo, 2mg, klonopin, bars, 1mg, xans, tolerance, ambien, clonazepam | 0.017 | 0.05 | 0.002 | 0.033 | -0.018 | <0.001 | -0.028 | -0.008 | 0.008 | 0.773 | -0.029 | 0.045 | 0.019 | 0.253 | -0.011 | 0.049 |
| 41 | lose, pounds, lost, gained, healthy, overweight, lbs, losing, gain, obese, fat, loss, bmi, gaining, weighed, skinny | 0.031 | <0.001 | 0.016 | 0.046 | 0.062 | <0.001 | 0.052 | 0.072 | 0.012 | 0.634 | -0.025 | 0.049 | 0.117 | <0.001 | 0.088 | 0.147 |
| 42 | fasting, diet, intermittent, keto, fast, carbs, omad, cico, results, dieting, exercising, diets, low carb, lifestyle, keto diet, window | 0.027 | 0.00 | 0.011 | 0.042 | 0.027 | <0.001 | 0.017 | 0.037 | -0.034 | 0.137 | -0.071 | 0.003 | 0.099 | <0.001 | 0.069 | 0.128 |
| 43 | times, again, many, tried, try, trying, past, failed, different, multiple, fail, attempts, attempt, cycle, stick, no matter | -0.005 | 0.61 | -0.020 | 0.011 | 0.049 | <0.001 | 0.039 | 0.059 | -0.042 | 0.063 | -0.079 | -0.005 | 0.119 | <0.001 | 0.090 | 0.149 |
| 44 | question, why, does, questions, answer, ask, says, title, asking, stupid, mean, sorry, answers, understand, explain, curious | 0.025 | 0.00 | 0.009 | 0.040 | -0.004 | 0.439 | -0.014 | 0.006 | 0.006 | 0.841 | -0.031 | 0.043 | -0.058 | <0.001 | -0.087 | -0.028 |
| 45 | video, videos, youtube, check, instagram, reddit, content, link, watch, show, facebook, channel, watching, posted, posts, follow | 0.009 | 0.31 | -0.006 | 0.025 | 0.013 | 0.021 | 0.002 | 0.023 | 0.033 | 0.153 | -0.004 | 0.070 | -0.047 | 0.004 | -0.076 | -0.017 |
| 46 | drugs, drug, harm, illegal, reduction, legal, users, dangerous, substances, society, war, opinion, against, substance, addicts, user | -0.015 | 0.09 | -0.031 | 0.000 | -0.011 | 0.038 | -0.021 | -0.001 | -0.030 | 0.196 | -0.067 | 0.007 | -0.065 | <0.001 | -0.094 | -0.035 |
| 47 | two, weeks, three, months, ago, week, four, month, past, five, six, couple, second, ten, seven, third | -0.026 | 0.00 | -0.041 | -0.010 | 0.004 | 0.491 | -0.006 | 0.014 | -0.078 | <0.001 | -0.114 | -0.041 | 0.042 | 0.009 | 0.012 | 0.072 |
| 48 | someone, need, please, who, anyone, talk, tell, support, send, wants, knows, buddy, needs, interested, message, willing | -0.008 | 0.37 | -0.024 | 0.007 | 0.023 | <0.001 | 0.013 | 0.034 | -0.001 | 0.968 | -0.038 | 0.036 | 0.033 | 0.043 | 0.003 | 0.063 |
| 49 | rehab, detox, treatment, hospital, program, therapy, center, therapist, insurance, recovery, medical, appointment, relapsed, inpatient, outpatient, relapse | -0.005 | 0.64 | -0.020 | 0.011 | 0.033 | <0.001 | 0.023 | 0.044 | -0.078 | <0.001 | -0.115 | -0.042 | 0.057 | <0.001 | 0.027 | 0.086 |
| 50 | sleep, night, hours, bed, sleeping, wake up, tired, morning, fall asleep, awake, nights, slept, hour, insomnia, waking, melatonin | 0.005 | 0.59 | -0.010 | 0.021 | 0.009 | 0.115 | -0.001 | 0.019 | -0.039 | 0.089 | -0.076 | -0.002 | 0.080 | <0.001 | 0.051 | 0.110 |
| 51 | him, his, friend, said, told, guy, asked, hes, he's, mine, gave, call, himself, met, buddy, dude | -0.045 | <0.001 | -0.061 | -0.030 | -0.020 | <0.001 | -0.030 | -0.010 | -0.036 | 0.115 | -0.073 | 0.001 | 0.026 | 0.117 | -0.004 | 0.055 |
| 52 | relationship, together, boyfriend, partner, ex, dating, broke, met, moved, date, relationships, girl, ended, loved, girlfriend, breakup | -0.036 | <0.001 | -0.052 | -0.021 | 0.042 | <0.001 | 0.032 | 0.052 | -0.103 | <0.001 | -0.139 | -0.066 | 0.108 | <0.001 | 0.078 | 0.137 |
| 53 | order, online, ordered, shipping, package, site, website, mail, email, sent, ordering, ship, send, received, delivery, company | 0.013 | 0.14 | -0.003 | 0.028 | -0.018 | 0.001 | -0.028 | -0.008 | 0.031 | 0.180 | -0.006 | 0.068 | -0.067 | <0.001 | -0.096 | -0.037 |
| 54 | eat, breakfast, lunch, protein, dinner, eggs, fruit, milk, chicken, bread, meal, snack, rice, salad, banana, cheese | 0.013 | 0.14 | -0.002 | 0.029 | -0.020 | <0.001 | -0.030 | -0.010 | 0.017 | 0.511 | -0.020 | 0.054 | 0.000 | 0.993 | -0.030 | 0.030 |
| 55 | bike, tire, frame, rack, wheel, tires, seat, tube, front, lock, stem, fork, mount, flat, rear, saddle | 0.009 | 0.32 | -0.006 | 0.025 | -0.019 | <0.001 | -0.029 | -0.009 | 0.194 | <0.001 | 0.158 | 0.229 | -0.090 | <0.001 | -0.119 | -0.060 |
| 56 | id, ill, havent, thats, sure, cant, didnt, isnt, theres, probably, wont, doesnt, wasnt, guess, wouldnt, yet | 0.035 | <0.001 | 0.019 | 0.050 | 0.041 | <0.001 | 0.031 | 0.051 | -0.029 | 0.215 | -0.066 | 0.008 | 0.040 | 0.013 | 0.010 | 0.070 |
| 57 | here, sub, thank, post, everyone, posts, reading, community, stories, read, hope, support, reddit, share, subreddit, posting | -0.036 | <0.001 | -0.051 | -0.020 | 0.031 | <0.001 | 0.021 | 0.041 | -0.016 | 0.521 | -0.053 | 0.021 | 0.026 | 0.112 | -0.004 | 0.056 |
| 58 | find, anyone, online, does, information, found, info, google, seem, search, internet, finding, anywhere, answer, searching, research | 0.003 | 0.77 | -0.012 | 0.019 | -0.005 | 0.372 | -0.015 | 0.005 | 0.026 | 0.267 | -0.011 | 0.063 | -0.055 | <0.001 | -0.084 | -0.025 |
| 59 | quit, smoking, quitting, smoke, cigarettes, cravings, smoker, smoked, cigarette, cold turkey, pack, attempt, habit, date, chantix, failed | -0.018 | 0.04 | -0.034 | -0.003 | 0.041 | <0.001 | 0.031 | 0.051 | -0.062 | 0.005 | -0.099 | -0.025 | 0.150 | <0.001 | 0.121 | 0.179 |
| 60 | feeling, tired, energy, normal, brain, depressed, mood, anxious, fog, physically, mentally, depression, exhausted, anyone else, motivation, irritable | -0.006 | 0.49 | -0.022 | 0.009 | 0.038 | <0.001 | 0.028 | 0.048 | -0.038 | 0.097 | -0.075 | -0.001 | 0.141 | <0.001 | 0.112 | 0.170 |
| 61 | bit, little, pretty, too, maybe, kind, stuff, though, sure, actually, guess, might, quite, probably, kinda, anyway | -0.006 | 0.54 | -0.021 | 0.010 | -0.005 | 0.415 | -0.015 | 0.005 | -0.033 | 0.158 | -0.070 | 0.004 | 0.003 | 0.881 | -0.027 | 0.033 |
| 62 | buy, cbd, online, cannabis, seeds, thc, plant, marijuana, oil, grow, legal, plants, growing, strain, uk, australia | 0.030 | <0.001 | 0.015 | 0.046 | -0.023 | <0.001 | -0.033 | -0.013 | 0.049 | 0.030 | 0.012 | 0.086 | -0.069 | <0.001 | -0.099 | -0.040 |
| 63 | pain, left, side, arm, hurt, legs, sore, leg, lower, hurts, neck, shoulder, feet, knees, chest, muscles | 0.024 | 0.00 | 0.009 | 0.040 | 0.011 | 0.056 | 0.000 | 0.021 | 0.005 | 0.855 | -0.032 | 0.042 | 0.063 | <0.001 | 0.033 | 0.093 |
| 64 | scale, weigh, water, weighed, number, gained, seeing, gain, scales, weighing, results, pound, numbers, discouraged, stepped, frustrated | 0.032 | <0.001 | 0.016 | 0.047 | 0.020 | <0.001 | 0.010 | 0.030 | -0.005 | 0.855 | -0.042 | 0.032 | 0.070 | <0.001 | 0.040 | 0.100 |
| 65 | bike, chain, shimano, brakes, speed, brake, front, cassette, gear, rear, gears, crank, wheel, disc, shifters, parts | -0.001 | 0.96 | -0.016 | 0.015 | -0.017 | 0.001 | -0.027 | -0.007 | 0.148 | <0.001 | 0.111 | 0.184 | -0.086 | <0.001 | -0.116 | -0.057 |
| 66 | work, home, hours, morning, hour, walk, drive, bed, tomorrow, pm, early, leave, wake up, late, minutes, evening | -0.014 | 0.12 | -0.029 | 0.002 | -0.026 | <0.001 | -0.036 | -0.016 | -0.060 | 0.008 | -0.096 | -0.023 | 0.040 | 0.012 | 0.011 | 0.070 |
| 67 | dxm, kratom, phenibut, dph, dose, benadryl, ghb, syrup, cough, nutmeg, safe, mg, grams, combo, doses, codeine | 0.017 | 0.06 | 0.001 | 0.032 | -0.033 | <0.001 | -0.043 | -0.023 | -0.073 | <0.001 | -0.110 | -0.037 | -0.026 | 0.112 | -0.056 | 0.004 |
| 68 | night, morning, today, feeling, woke up, bed, hangover, iwndwyt, yesterday, hungover, wake up, waking, tomorrow, early, drank, saturday | -0.041 | <0.001 | -0.056 | -0.025 | 0.015 | 0.006 | 0.005 | 0.025 | -0.045 | 0.048 | -0.082 | -0.008 | 0.093 | <0.001 | 0.063 | 0.122 |
| 69 | goal, goals, set, plan, reach, term, achieve, setting, focus, step, hopefully, challenge, success, realistic, motivated, date | -0.014 | 0.11 | -0.030 | 0.001 | 0.034 | <0.001 | 0.024 | 0.044 | -0.045 | 0.047 | -0.082 | -0.008 | 0.079 | <0.001 | 0.049 | 0.109 |
| 70 | challenge, tonight, hello, trend, 30 day accountability, score, keeps, journals, grateful, express, pages, new recipe once, gratitude, fast food, journaling, treat | -0.030 | <0.001 | -0.046 | -0.015 | -0.038 | <0.001 | -0.048 | -0.028 | 0.034 | 0.145 | -0.003 | 0.071 | -0.004 | 0.856 | -0.034 | 0.026 |
| 71 | snorting, nose, snort, powder, pill, snorted, pills, oral, crush, mouth, taste, swallow, tongue, orally, iv, crushed | 0.030 | <0.001 | 0.015 | 0.046 | -0.017 | 0.001 | -0.027 | -0.007 | -0.069 | 0.002 | -0.106 | -0.032 | -0.025 | 0.130 | -0.055 | 0.005 |
| 72 | bag, pack, backpack, gear, backpacking, tent, carry, camping, bags, suggestions, recommendations, hiking, bring, dp, trips, travel | -0.009 | 0.34 | -0.024 | 0.007 | -0.027 | <0.001 | -0.037 | -0.017 | -0.044 | 0.053 | -0.080 | -0.007 | -0.101 | <0.001 | -0.130 | -0.071 |
| 73 | said, told, asked, saying, talking, yes, telling, called, conversation, oh, asking, comments, mentioned, comment, yeah, talked | -0.059 | <0.001 | -0.075 | -0.044 | -0.018 | <0.001 | -0.028 | -0.008 | -0.014 | 0.576 | -0.051 | 0.023 | 0.031 | 0.054 | 0.002 | 0.061 |
| 74 | parents, house, went, home, mom, told, didnt, said, dad, room, remember, came, called, ended up, brother, phone | -0.023 | 0.01 | -0.039 | -0.008 | -0.010 | 0.086 | -0.020 | 0.001 | -0.045 | 0.047 | -0.082 | -0.008 | 0.071 | <0.001 | 0.041 | 0.100 |
| 75 | pod, pods, vape, device, caliburn, juice, juul, coils, vaping, pod system, flavor, coil, smok, nord, mtl, novo | 0.024 | 0.01 | 0.009 | 0.040 | -0.025 | <0.001 | -0.035 | -0.015 | 0.063 | 0.004 | 0.026 | 0.100 | -0.111 | <0.001 | -0.140 | -0.082 |
| 76 | didn't, i'd, wasn't, thought, felt, couldn't, knew, wanted, that's, decided, wouldn't, came, realized, haven't, needed, ended up | -0.052 | <0.001 | -0.068 | -0.037 | -0.020 | <0.001 | -0.030 | -0.010 | -0.032 | 0.166 | -0.069 | 0.005 | 0.061 | <0.001 | 0.031 | 0.090 |
| 77 | run, running, miles, training, mile, race, ran, marathon, walk, trail, runs, walking, 5k, distance, minutes, pace | 0.020 | 0.03 | 0.004 | 0.035 | -0.011 | 0.039 | -0.021 | -0.001 | 0.013 | 0.603 | -0.024 | 0.050 | 0.038 | 0.018 | 0.008 | 0.068 |
| 78 | cannabis, market, poll, view, world, industry, 2020, growth, news, business, company, marijuana, global, american, analysis, island | 0.026 | 0.00 | 0.011 | 0.042 | -0.007 | 0.190 | -0.017 | 0.003 | 0.001 | 0.961 | -0.036 | 0.038 | -0.076 | <0.001 | -0.106 | -0.047 |
| 79 | bike, nbd, gravel, road, tires, wheels, ride, carbon, tire, riding, disc, specialized, frame, 2020, tubeless, diverge | 0.047 | <0.001 | 0.031 | 0.062 | -0.010 | 0.073 | -0.020 | 0.000 | 0.220 | <0.001 | 0.185 | 0.255 | -0.048 | 0.003 | -0.078 | -0.019 |
| 80 | thank, iwndwyt, support, sub, everyone, sobriety, community, proud, journey, grateful, 100, amazing, helped, stories, sd, thankful | -0.032 | <0.001 | -0.048 | -0.017 | 0.046 | <0.001 | 0.036 | 0.056 | -0.021 | 0.383 | -0.058 | 0.016 | 0.083 | <0.001 | 0.053 | 0.112 |
| 81 | youre, thats, cant, doesnt, ill, theres, isnt, id, wont, yourself, youve, theyre, youll, whats, arent, wouldnt | 0.019 | 0.03 | 0.004 | 0.035 | 0.037 | <0.001 | 0.027 | 0.047 | -0.019 | 0.448 | -0.056 | 0.018 | 0.069 | <0.001 | 0.039 | 0.098 |
| 82 | calories, eating, lose, losing, pounds, loss, 1200, exercise, plateau, per, deficit, maintenance, gain, cals, calorie, stuck | 0.070 | <0.001 | 0.055 | 0.086 | 0.030 | <0.001 | 0.020 | 0.040 | 0.014 | 0.586 | -0.023 | 0.051 | 0.093 | <0.001 | 0.063 | 0.122 |
| 83 | eat, food, eating, hungry, meal, full, dinner, hunger, ate, meals, appetite, lunch, breakfast, snack, stomach, snacks | 0.025 | 0.00 | 0.010 | 0.041 | 0.004 | 0.447 | -0.006 | 0.014 | -0.018 | 0.491 | -0.055 | 0.019 | 0.107 | <0.001 | 0.078 | 0.137 |
| 84 | felt, happened, bed, hospital, woke up, die, couldnt, couldn't, heart, shaking, dying, passed, breathing, ambulance, bathroom, seizure | -0.015 | 0.10 | -0.030 | 0.001 | -0.024 | <0.001 | -0.034 | -0.014 | -0.033 | 0.151 | -0.070 | 0.004 | 0.118 | <0.001 | 0.088 | 0.147 |
| 85 | why, cant, thats, makes, doesnt, hate, saying, mean, literally, stupid, isnt, theyre, understand, theres, rant, annoying | -0.013 | 0.13 | -0.029 | 0.002 | 0.033 | <0.001 | 0.023 | 0.043 | -0.011 | 0.675 | -0.048 | 0.026 | 0.039 | 0.016 | 0.009 | 0.068 |
| 86 | brain, effects, dopamine, damage, effect, increase, serotonin, system, levels, receptors, memory, chemical, cognitive, causes, natural, wiki | 0.024 | 0.01 | 0.009 | 0.040 | 0.002 | 0.666 | -0.008 | 0.013 | 0.022 | 0.362 | -0.015 | 0.059 | 0.013 | 0.438 | -0.017 | 0.043 |
| 87 | withdrawal, symptoms, withdrawals, taper, cold turkey, tapering, detox, physical, shakes, seizures, seizure, worst, shaking, insomnia, sweating, severe | 0.011 | 0.21 | -0.004 | 0.027 | 0.026 | <0.001 | 0.016 | 0.036 | -0.061 | 0.006 | -0.098 | -0.024 | 0.125 | <0.001 | 0.095 | 0.154 |
| 88 | who, their, different, same, someone, why, person, those, others, seen, they're, seems, doesn't, that's, seem, there's | -0.020 | 0.02 | -0.036 | -0.005 | 0.010 | 0.061 | 0.000 | 0.020 | -0.051 | 0.025 | -0.088 | -0.014 | -0.035 | 0.029 | -0.065 | -0.006 |
| 89 | mind, reality, world, universe, god, dmt, sense, state, consciousness, human, death, psychedelics, spiritual, words, existence, earth | -0.009 | 0.32 | -0.025 | 0.006 | -0.006 | 0.268 | -0.016 | 0.004 | -0.050 | 0.027 | -0.087 | -0.013 | 0.042 | 0.010 | 0.012 | 0.071 |
| 90 | cant, stop, need, ill, anymore, doesnt, wont, seem, thats, isnt, havent, hate, theres, scared, whats, couldnt | 0.025 | 0.00 | 0.010 | 0.041 | 0.058 | <0.001 | 0.048 | 0.068 | 0.013 | 0.602 | -0.024 | 0.050 | 0.089 | <0.001 | 0.060 | 0.119 |
| 91 | depression, anxiety, mental, mental health, issues, disorder, medication, diagnosed, self, therapy, illness, depressed, ptsd, severe, meds, psychosis | 0.014 | 0.11 | -0.001 | 0.030 | 0.032 | <0.001 | 0.022 | 0.042 | -0.049 | 0.030 | -0.086 | -0.012 | 0.075 | <0.001 | 0.045 | 0.104 |
| 92 | sober, sobriety, months, year, today, iwndwyt, month, stay, proud, longest, 30, grateful, birthday, booze, relapse, staying | -0.017 | 0.06 | -0.032 | -0.001 | 0.055 | <0.001 | 0.045 | 0.065 | -0.070 | 0.001 | -0.107 | -0.033 | 0.114 | <0.001 | 0.085 | 0.143 |
| 93 | their, however, may, such, fact, simply, given, must, cannot, situation, case, whether, likely, several, large, despite | 0.005 | 0.57 | -0.010 | 0.021 | 0.011 | 0.049 | 0.001 | 0.021 | -0.015 | 0.574 | -0.051 | 0.023 | -0.039 | 0.014 | -0.069 | -0.010 |
| 94 | test, drug, pass, urine, tested, tests, testing, system, hair, positive, clean, show, kit, thc, piss, lab | -0.007 | 0.48 | -0.022 | 0.009 | -0.040 | <0.001 | -0.050 | -0.030 | 0.029 | 0.211 | -0.008 | 0.066 | -0.022 | 0.174 | -0.052 | 0.007 |
| 95 | battery, coil, mod, device, review, button, power, kit, mode, tank, coils, fire, box, pod, wattage, screen | -0.027 | 0.00 | -0.043 | -0.012 | -0.057 | <0.001 | -0.067 | -0.047 | 0.114 | <0.001 | 0.077 | 0.150 | -0.134 | <0.001 | -0.163 | -0.105 |
| 96 | weed, smoke, smoking, smoked, high, joint, bong, marijuana, pot, bowl, joints, stoned, blunt, tobacco, hash, cannabis | 0.018 | 0.04 | 0.003 | 0.034 | -0.041 | <0.001 | -0.051 | -0.031 | -0.046 | 0.043 | -0.083 | -0.009 | 0.140 | <0.001 | 0.111 | 0.169 |
| 97 | vaping, electronic cigarette, comments, chart, wish, contests, others, questions, knew, related, comparison, decision, courtesy, size, 22, join | 0.007 | 0.47 | -0.009 | 0.022 | 0.004 | 0.524 | -0.006 | 0.014 | 0.003 | 0.936 | -0.034 | 0.040 | -0.077 | <0.001 | -0.107 | -0.048 |
| 98 | eating, binge, food, healthy, disorder, habits, binging, gained, gain, relationship, unhealthy, cycle, struggled, ed, gaining, emotional | 0.042 | <0.001 | 0.026 | 0.057 | 0.063 | <0.001 | 0.052 | 0.073 | 0.005 | 0.865 | -0.032 | 0.042 | 0.156 | <0.001 | 0.127 | 0.185 |
| 99 | shit, fucking, fuck, fucked, ass, man, damn, gonna, holy, oh, god, yeah, literally, hell, stupid, lol | -0.028 | 0.00 | -0.043 | -0.012 | -0.024 | <0.001 | -0.034 | -0.014 | 0.001 | 0.975 | -0.036 | 0.038 | 0.013 | 0.434 | -0.016 | 0.043 |
| 100 | muscle, gym, workout, training, gain, cardio, build, lifting, mass, routine, strength, muscles, protein, workouts, gains, building | 0.034 | <0.001 | 0.019 | 0.050 | -0.004 | 0.491 | -0.014 | 0.006 | -0.032 | 0.168 | -0.069 | 0.005 | 0.020 | 0.233 | -0.010 | 0.049 |
| 101 | #x200b, question, redd, post, small, that's, fine, ask, png, welcome, minor, resources, flair, faq, q , ask questions | -0.128 | <0.001 | -0.143 | -0.113 | -0.108 | <0.001 | -0.117 | -0.098 | -0.078 | <0.001 | -0.114 | -0.041 | -0.108 | <0.001 | -0.138 | -0.079 |
| 102 | music, game, play, watch, song, playing, watching, movie, listen, listening, games, show, movies, songs, tv, favorite | -0.009 | 0.33 | -0.024 | 0.007 | -0.023 | <0.001 | -0.033 | -0.013 | 0.013 | 0.600 | -0.024 | 0.050 | -0.025 | 0.133 | -0.054 | 0.005 |
| 103 | mdma, molly, taking, roll, pill, rolling, ecstasy, 2cb, pills, festival, comedown, md, dose, rave, party, planning | 0.002 | 0.88 | -0.014 | 0.017 | -0.069 | <0.001 | -0.079 | -0.059 | -0.052 | 0.021 | -0.089 | -0.015 | -0.004 | 0.861 | -0.033 | 0.026 |
| 104 | today, tomorrow, iwndwyt, tonight, yesterday, proud, hope, excited, longest, tough, wish me luck, rough, checking, glad, stay strong, looking forward | -0.037 | <0.001 | -0.053 | -0.022 | 0.028 | <0.001 | 0.018 | 0.038 | -0.037 | 0.106 | -0.074 | 0.000 | 0.080 | <0.001 | 0.050 | 0.110 |
| 105 | friends, social, alone, fun, talk, group, hang, lonely, meet, party, socially, parties, awkward, anxious, hanging, comfortable | -0.033 | <0.001 | -0.049 | -0.018 | 0.006 | 0.335 | -0.004 | 0.016 | -0.080 | <0.001 | -0.116 | -0.043 | 0.099 | <0.001 | 0.069 | 0.128 |
| 106 | trip, acid, lsd, bad, tripping, tabs, tab, trips, shrooms, tripped, psychedelics, visuals, intense, drop, dropped, psychedelic | -0.020 | 0.03 | -0.035 | -0.004 | -0.052 | <0.001 | -0.062 | -0.042 | -0.149 | <0.001 | -0.185 | -0.113 | 0.053 | <0.001 | 0.023 | 0.083 |
| 107 | #x200b, reviews, review, cons, pros, , device, flow, gallery, air, , ensure, lets, , solid, liquid | -0.095 | <0.001 | -0.111 | -0.080 | -0.075 | <0.001 | -0.085 | -0.065 | -0.015 | 0.545 | -0.052 | 0.022 | -0.127 | <0.001 | -0.156 | -0.097 |
| 108 | 2019, since, year, 2020, 2018, march, january, june, december, may, august, april, july, february, 2017, 1st | 0.015 | 0.09 | 0.000 | 0.031 | 0.041 | <0.001 | 0.031 | 0.051 | -0.045 | 0.048 | -0.082 | -0.008 | 0.044 | 0.006 | 0.015 | 0.074 |
| 109 | shrooms, lsd, trip, mushrooms, dmt, psychedelics, dose, acid, psychedelic, grams, tripping, psilocybin, mushroom, microdosing, shroom, tabs | 0.018 | 0.04 | 0.002 | 0.033 | -0.044 | <0.001 | -0.054 | -0.034 | -0.146 | <0.001 | -0.182 | -0.109 | -0.005 | 0.808 | -0.035 | 0.025 |
| 110 | anyone, experience, does, wondering, hear, experiences, curious, similar, heard, thoughts, stories, share, anyone else, interested, opinions, anybody | 0.009 | 0.35 | -0.007 | 0.024 | -0.005 | 0.378 | -0.015 | 0.005 | -0.038 | 0.099 | -0.075 | -0.001 | -0.030 | 0.065 | -0.060 | 0.000 |
| 111 | beer, wine, bottle, iwndwyt, glass, tonight, beers, bottles, dinner, booze, store, glass of wine, fridge, husband, poured, red | -0.031 | <0.001 | -0.047 | -0.016 | 0.029 | <0.001 | 0.019 | 0.040 | -0.010 | 0.689 | -0.047 | 0.027 | 0.015 | 0.367 | -0.015 | 0.045 |
| 112 | every, drinks, stop, problem, once, beer, beers, drunk, weekends, weekend, usually, sometimes, nights, binge, drank, cut | -0.007 | 0.47 | -0.022 | 0.009 | 0.044 | <0.001 | 0.034 | 0.054 | -0.094 | <0.001 | -0.131 | -0.057 | 0.119 | <0.001 | 0.090 | 0.148 |
| 113 | change, finally, journey, health, proud, changes, lifestyle, mentally, ready, motivation, physically, excited, self, healthier, physical, filler | -0.014 | 0.11 | -0.030 | 0.001 | 0.064 | <0.001 | 0.054 | 0.074 | -0.004 | 0.873 | -0.041 | 0.033 | 0.122 | <0.001 | 0.093 | 0.152 |
| 114 | please, questions, thread, message, ask, subreddit, check, weekly, discussion, posted, rules, answer, information, participating, monday, report | -0.010 | 0.28 | -0.025 | 0.006 | -0.013 | 0.020 | -0.023 | -0.003 | -0.006 | 0.836 | -0.043 | 0.031 | -0.031 | 0.053 | -0.061 | -0.002 |
| 115 | size, fit, clothes, wear, pants, wearing, jeans, dress, shirt, sizes, tight, shirts, smaller, fitting, belt, nsv | -0.038 | <0.001 | -0.054 | -0.023 | -0.008 | 0.156 | -0.018 | 0.002 | 0.049 | 0.032 | 0.012 | 0.085 | -0.074 | <0.001 | -0.103 | -0.044 |
| 116 | anxiety, panic, attack, attacks, anxious, heart, scared, fear, calm, chest, afraid, paranoid, paranoia, nervous, racing, disorder | 0.000 | 0.97 | -0.016 | 0.015 | 0.004 | 0.439 | -0.006 | 0.014 | -0.060 | 0.007 | -0.097 | -0.023 | 0.100 | <0.001 | 0.070 | 0.129 |
| 117 | battery, mod, batteries, mods, cell, mech, cells, charge, charger, voltage, 18650, current, testing, rating, power, dual | -0.077 | <0.001 | -0.092 | -0.061 | -0.071 | <0.001 | -0.081 | -0.061 | 0.058 | 0.008 | 0.022 | 0.095 | -0.118 | <0.001 | -0.147 | -0.088 |
| 118 | didnt, wasnt, id, felt, did, couldnt, thought, wanted, knew, thats, wouldnt, realized, needed, came, kept, havent | -0.003 | 0.79 | -0.018 | 0.013 | 0.017 | 0.002 | 0.007 | 0.027 | -0.036 | 0.115 | -0.073 | 0.001 | 0.092 | <0.001 | 0.063 | 0.122 |
| 119 | play, college, sports, active, shape, high school, playing, football, team, swim, kid, basketball, played, swimming, sport, game | -0.023 | 0.01 | -0.038 | -0.007 | 0.000 | 0.988 | -0.010 | 0.010 | -0.022 | 0.362 | -0.059 | 0.015 | 0.053 | <0.001 | 0.024 | 0.083 |
| 120 | self, others, feelings, emotional, learned, learning, learn, fear, emotions, grateful, positive, sense, negative, happiness, present, accept | -0.013 | 0.14 | -0.029 | 0.002 | 0.043 | <0.001 | 0.032 | 0.053 | -0.034 | 0.137 | -0.071 | 0.003 | 0.082 | <0.001 | 0.053 | 0.112 |
| 121 | free, our, sale, code, products, giveaway, check, win, enter, product, discount, winners, must, site, offer, shipping | -0.028 | <0.001 | -0.044 | -0.013 | -0.014 | 0.008 | -0.024 | -0.004 | 0.013 | 0.602 | -0.024 | 0.050 | -0.067 | <0.001 | -0.097 | -0.037 |
| 122 | restrict sr, motivation, automoderator, share, running, what's, thread, track, nsv, monday, below, whats, q , related, keeping, thursday | -0.075 | <0.001 | -0.091 | -0.060 | -0.048 | <0.001 | -0.058 | -0.038 | 0.050 | 0.029 | 0.013 | 0.086 | -0.032 | 0.050 | -0.062 | -0.002 |
| 123 | world, dark, eyes, soul, fear, deep, death, monster, escape, voice, sun, fire, broken, demon, filled, darkness | -0.030 | <0.001 | -0.046 | -0.015 | 0.014 | 0.012 | 0.004 | 0.024 | -0.019 | 0.452 | -0.056 | 0.018 | 0.050 | 0.002 | 0.020 | 0.080 |
| 124 | ski, skiing, skis, season, snow, mountain, pass, powder, pair, skier, resort, bindings, resorts, winter, boots, east | 0.013 | 0.14 | -0.003 | 0.028 | -0.041 | <0.001 | -0.051 | -0.031 | -0.108 | <0.001 | -0.145 | -0.072 | -0.066 | <0.001 | -0.096 | -0.037 |
| 125 | feeling, happy, sad, miss, depressed, makes, feels, anymore, depression, hate, alone, happiness, feelings, wish, world, lonely | -0.017 | 0.05 | -0.033 | -0.002 | 0.061 | <0.001 | 0.051 | 0.071 | -0.029 | 0.215 | -0.066 | 0.008 | 0.111 | <0.001 | 0.081 | 0.140 |
| 126 | tank, coils, mod, coil, mesh, vape, rta, rda, mini, tanks, vaping, flavor, build, wire, kit, mtl | 0.019 | 0.03 | 0.003 | 0.034 | -0.026 | <0.001 | -0.036 | -0.016 | 0.083 | <0.001 | 0.046 | 0.119 | -0.135 | <0.001 | -0.164 | -0.106 |
| 127 | guys, hey, thanks, wondering, hi, advance, suggestions, tips, yall, hello, ideas, appreciated, recommendations, appreciate, recommend, fellow | 0.004 | 0.67 | -0.011 | 0.020 | -0.017 | 0.001 | -0.028 | -0.007 | -0.019 | 0.454 | -0.056 | 0.018 | -0.054 | <0.001 | -0.083 | -0.024 |
| 128 | school, college, class, high school, summer, university, classes, semester, parents, student, studying, study, teacher, degree, grade, grades | -0.043 | <0.001 | -0.059 | -0.028 | -0.017 | 0.002 | -0.027 | -0.007 | -0.085 | <0.001 | -0.122 | -0.048 | 0.046 | 0.004 | 0.016 | 0.076 |
| 129 | can't, doesn't, something, why, that's, anything, isn't, didn't, problem, seem, i'll, there's, won't, nothing, seems, wrong | -0.034 | <0.001 | -0.049 | -0.018 | 0.020 | <0.001 | 0.010 | 0.030 | -0.020 | 0.416 | -0.057 | 0.017 | 0.015 | 0.386 | -0.015 | 0.044 |
| 130 | nicotine, vaping, smoking, quit, vape, cigarettes, quitting, juul, tobacco, cigarette, gum, cigs, patch, patches, addicted, pack | -0.001 | 0.90 | -0.017 | 0.014 | 0.008 | 0.140 | -0.002 | 0.018 | 0.012 | 0.625 | -0.025 | 0.049 | 0.026 | 0.109 | -0.003 | 0.056 |
| 131 | add, oil, water, recipe, minutes, cup, salt, cook, heat, ingredients, pan, garlic, onion, mix, sauce, pepper | 0.031 | <0.001 | 0.015 | 0.046 | -0.031 | <0.001 | -0.041 | -0.021 | -0.017 | 0.511 | -0.054 | 0.020 | -0.081 | <0.001 | -0.110 | -0.051 |
| 132 | alcohol, problem, alcoholic, drunk, drank, alcoholism, sobriety, drinks, booze, drinker, control, relationship, problems, binge, moderation, alcoholics | -0.003 | 0.79 | -0.018 | 0.013 | 0.062 | <0.001 | 0.052 | 0.072 | -0.046 | 0.041 | -0.083 | -0.009 | 0.126 | <0.001 | 0.096 | 0.155 |
| 133 | him, his, hes, he's, boyfriend, husband, dad, brother, himself, says, wants, doesnt, gets, needs, bf, father | -0.018 | 0.05 | -0.033 | -0.002 | 0.010 | 0.075 | 0.000 | 0.020 | -0.035 | 0.131 | -0.072 | 0.002 | 0.039 | 0.017 | 0.009 | 0.068 |
| 134 | down, hit, bottom, rock, turn, fall, top, hole, path, slow, line, falling, edge, fell, direction, hitting | -0.011 | 0.24 | -0.026 | 0.005 | 0.005 | 0.421 | -0.005 | 0.015 | -0.033 | 0.150 | -0.070 | 0.004 | 0.013 | 0.434 | -0.016 | 0.043 |
| 135 | marijuana, vaping, state, cannabis, medical, products, ban, tobacco, legal, bill, legalization, law, fda, states, news, sales | -0.017 | 0.05 | -0.033 | -0.002 | -0.021 | <0.001 | -0.031 | -0.011 | 0.024 | 0.319 | -0.013 | 0.061 | -0.090 | <0.001 | -0.120 | -0.061 |
| 136 | alcohol, night, bottle, drank, drunk, vodka, beer, wine, beers, whiskey, shots, bottles, liquor, alcoholic, drinks, booze | -0.025 | 0.00 | -0.040 | -0.009 | 0.040 | <0.001 | 0.030 | 0.050 | -0.080 | <0.001 | -0.116 | -0.043 | 0.051 | 0.001 | 0.021 | 0.080 |
| 137 | bike, bikes, road, frame, price, worth, html, model, trek, buying, brand, vs, shop, carbon, giant, disc | -0.009 | 0.33 | -0.024 | 0.007 | -0.031 | <0.001 | -0.041 | -0.021 | 0.149 | <0.001 | 0.113 | 0.185 | -0.116 | <0.001 | -0.145 | -0.086 |
| 138 | drugs, weed, drug, mdma, lsd, coke, ketamine, shrooms, acid, xanax, cocaine, molly, psychedelics, benzos, substances, adderall | 0.018 | 0.04 | 0.003 | 0.034 | -0.039 | <0.001 | -0.049 | -0.029 | -0.074 | <0.001 | -0.110 | -0.037 | 0.090 | <0.001 | 0.060 | 0.119 |
| 139 | look, skin, loose, face, mirror, hair, looks, looked, pictures, marks, stretch, noticed, picture, photos, eyes, notice | 0.002 | 0.85 | -0.013 | 0.018 | 0.005 | 0.375 | -0.005 | 0.015 | 0.000 | 1.000 | -0.037 | 0.037 | 0.031 | 0.058 | 0.001 | 0.060 |
| 140 | stomach, pain, sick, nausea, issues, headache, headaches, anyone else, nauseous, vomiting, symptoms, reflux, ibs, throwing, diarrhea, empty | 0.017 | 0.05 | 0.002 | 0.033 | 0.014 | 0.008 | 0.004 | 0.024 | -0.026 | 0.267 | -0.063 | 0.011 | 0.078 | <0.001 | 0.049 | 0.108 |
| 141 | backpack, ride, road, trail, cycling, hike, hiking, rides, mountain, miles, gravel, bicycle, city, roads, trails, hybrid | 0.011 | 0.22 | -0.004 | 0.027 | -0.024 | <0.001 | -0.034 | -0.014 | 0.175 | <0.001 | 0.139 | 0.210 | -0.087 | <0.001 | -0.117 | -0.058 |
| 142 | adderall, vyvanse, ritalin, taking, adhd, mg, dose, prescribed, xr, tolerance, concerta, pills, stimulants, 30mg, methylphenidate, 20mg | -0.022 | 0.01 | -0.038 | -0.007 | -0.024 | <0.001 | -0.034 | -0.014 | 0.043 | 0.053 | 0.006 | 0.080 | -0.021 | 0.207 | -0.050 | 0.009 |
| 143 | new, share, daily, free, come, possible, picture, might, place, hopefully, questions, entire, comment, amp, input, 2020 | -0.081 | <0.001 | -0.096 | -0.066 | -0.063 | <0.001 | -0.073 | -0.053 | 0.058 | 0.009 | 0.021 | 0.095 | -0.049 | 0.002 | -0.079 | -0.019 |
| 144 | plan, daily, may, stick, free, hope, currently, here's, posting, space, reddit, top, track, taken, worry, means | -0.079 | <0.001 | -0.095 | -0.064 | -0.058 | <0.001 | -0.068 | -0.048 | 0.048 | 0.035 | 0.011 | 0.084 | -0.049 | 0.002 | -0.078 | -0.019 |
| 145 | juice, taste, flavor, flavors, vape, juices, sweet, liquid, tastes, menthol, fruit, bottles, strawberry, tobacco, brand, vg | 0.016 | 0.06 | 0.001 | 0.032 | -0.023 | <0.001 | -0.033 | -0.013 | 0.014 | 0.594 | -0.023 | 0.051 | -0.114 | <0.001 | -0.143 | -0.084 |
| 146 | light, battery, fire, fix, issue, charge, button, mod, charging, turn, smok, wont, pod, screen, firing, red | 0.026 | 0.00 | 0.010 | 0.041 | -0.016 | 0.002 | -0.026 | -0.006 | 0.153 | <0.001 | 0.117 | 0.189 | -0.113 | <0.001 | -0.142 | -0.084 |
| 147 | money, pay, spend, buy, spent, spending, saved, save, afford, bank, paid, card, cost, paying, account, cash | -0.039 | <0.001 | -0.055 | -0.024 | 0.003 | 0.608 | -0.007 | 0.013 | -0.067 | 0.002 | -0.103 | -0.030 | -0.005 | 0.791 | -0.035 | 0.024 |
| 148 | take, tramadol, took, codeine, mg, gabapentin, dose, safe, pills, lyrica, tolerance, pregabalin, 300mg, 100mg, tablets, 50mg | 0.030 | <0.001 | 0.015 | 0.046 | -0.022 | <0.001 | -0.032 | -0.012 | -0.008 | 0.776 | -0.045 | 0.029 | -0.021 | 0.197 | -0.051 | 0.009 |
| 149 | i'd, advice, however, quite, seems, isn't, issue, suggestions, option, appreciated, options, fairly, appreciate, although, considering, rather | 0.008 | 0.40 | -0.008 | 0.023 | -0.005 | 0.378 | -0.015 | 0.005 | -0.035 | 0.137 | -0.071 | 0.002 | -0.049 | 0.002 | -0.079 | -0.020 |
| 150 | airflow, rda, top, build, flavor, deck, rta, cap, coil, review, air, mm, squonk, holes, wicking, cotton | 0.014 | 0.12 | -0.001 | 0.030 | -0.014 | 0.008 | -0.024 | -0.004 | 0.065 | 0.003 | 0.028 | 0.101 | -0.128 | <0.001 | -0.157 | -0.098 |
| 151 | family, dad, kids, mom, father, parents, mother, old, son, brother, died, wife, child, husband, children, daughter | -0.030 | <0.001 | -0.045 | -0.014 | 0.030 | <0.001 | 0.020 | 0.040 | -0.049 | 0.032 | -0.085 | -0.012 | 0.007 | 0.727 | -0.023 | 0.036 |
| 152 | fat, cut, muscle, cutting, bulk, bf , lean, bulking, abs, bf, lifting, mass, body fat, gain, estimate, bf | 0.038 | <0.001 | 0.022 | 0.053 | 0.009 | 0.093 | -0.001 | 0.019 | -0.023 | 0.343 | -0.060 | 0.014 | 0.003 | 0.881 | -0.027 | 0.033 |
| 153 | you're, that's, yourself, you've, you'll, i'll, there's, isn't, won't, doesn't, what's, they're, let's, here's, aren't, it'll | -0.006 | 0.54 | -0.021 | 0.010 | 0.022 | <0.001 | 0.012 | 0.032 | -0.041 | 0.070 | -0.078 | -0.004 | 0.038 | 0.018 | 0.008 | 0.068 |
| 154 | night, friends, bar, party, drunk, fun, drinks, beer, tonight, bars, beers, booze, club, event, concert, game | -0.071 | <0.001 | -0.087 | -0.056 | -0.056 | <0.001 | -0.066 | -0.045 | -0.078 | <0.001 | -0.115 | -0.041 | 0.053 | <0.001 | 0.024 | 0.083 |
| 155 | pills, oxy, oxycodone, opiates, pill, morphine, opioids, suboxone, hydrocodone, tolerance, mg, kratom, fentanyl, opioid, percocet, opiate | 0.020 | 0.02 | 0.005 | 0.036 | -0.006 | 0.295 | -0.016 | 0.004 | -0.031 | 0.180 | -0.068 | 0.006 | -0.007 | 0.707 | -0.037 | 0.023 |
| 156 | speed, amphetamine, nitrous, balloon, amphetamines, paste, line, nitrous oxide, whippets, nos, balloons, alot, iam, lines, comedown, pure | -0.078 | <0.001 | -0.094 | -0.063 | -0.055 | <0.001 | -0.065 | -0.045 | 0.061 | 0.006 | 0.024 | 0.098 | -0.055 | <0.001 | -0.084 | -0.025 |
| 157 | between, higher, low, vs, lower, level, difference, average, compared, range, quality, above, slightly, differences, example, result | 0.037 | <0.001 | 0.021 | 0.052 | -0.004 | 0.456 | -0.014 | 0.006 | 0.031 | 0.180 | -0.006 | 0.068 | -0.060 | <0.001 | -0.089 | -0.030 |
| 158 | addiction, addicted, addict, drugs, drug, clean, become, addictions, addicts, addictive, substance, cocaine, substances, recovery, habit, relapse | -0.008 | 0.37 | -0.024 | 0.007 | 0.056 | <0.001 | 0.046 | 0.066 | -0.028 | 0.231 | -0.065 | 0.009 | 0.113 | <0.001 | 0.084 | 0.142 |
| 159 | dream, remember, dreams, weird, happened, memory, real, woke up, anyone else, wake up, crazy, memories, experienced, nightmares, dreaming, strange | -0.013 | 0.15 | -0.028 | 0.003 | 0.017 | 0.001 | 0.007 | 0.027 | -0.044 | 0.051 | -0.081 | -0.007 | 0.053 | <0.001 | 0.023 | 0.082 |
| 160 | throat, sick, chest, lungs, cough, breathing, breath, anyone else, symptoms, coughing, cold, breathe, experienced, flu, sore, asthma | 0.019 | 0.03 | 0.003 | 0.034 | 0.006 | 0.267 | -0.004 | 0.016 | -0.010 | 0.710 | -0.047 | 0.027 | 0.097 | <0.001 | 0.068 | 0.127 |
| 161 | use, drugs, drug, using, effects, used, substances, term, substance, side, recreational, often, safe, abuse, cocaine, usage | 0.024 | 0.01 | 0.009 | 0.040 | -0.006 | 0.334 | -0.016 | 0.004 | 0.028 | 0.237 | -0.010 | 0.064 | -0.061 | <0.001 | -0.090 | -0.031 |
| 162 | wanna, gonna, idk, shit, kinda, bc, lol, cause, cuz, yall, tho, rn, anyways, yeah, alot, lil | 0.046 | <0.001 | 0.031 | 0.062 | 0.006 | 0.297 | -0.004 | 0.016 | 0.012 | 0.634 | -0.025 | 0.049 | 0.036 | 0.026 | 0.006 | 0.066 |
| 163 | calories, food, eating, loss, track, tracking, cico, goal, exercise, calorie, foods, scale, meals, ate, habits, maintenance | 0.028 | 0.00 | 0.013 | 0.044 | 0.048 | <0.001 | 0.038 | 0.058 | -0.014 | 0.594 | -0.051 | 0.023 | 0.113 | <0.001 | 0.083 | 0.142 |
| 164 | exercise, cardio, workout, gym, walking, walk, minutes, workouts, hour, routine, running, weights, min, treadmill, hiit, minute | 0.045 | <0.001 | 0.030 | 0.061 | -0.005 | 0.366 | -0.015 | 0.005 | 0.002 | 0.960 | -0.035 | 0.039 | 0.088 | <0.001 | 0.058 | 0.117 |
| 165 | during, covid, home, quarantine, due, lockdown, baby, pandemic, pregnant, virus, closed, corona, pregnancy, coronavirus, march, staying | 0.209 | <0.001 | 0.194 | 0.224 | 0.209 | <0.001 | 0.199 | 0.219 | 0.165 | <0.001 | 0.128 | 0.200 | 0.231 | <0.001 | 0.202 | 0.259 |
| 166 | room, door, came, looked, saw, bed, face, walked, sat, floor, turned, open, hand, sitting, eyes, outside | -0.032 | <0.001 | -0.047 | -0.016 | -0.038 | <0.001 | -0.048 | -0.028 | -0.019 | 0.448 | -0.056 | 0.018 | 0.023 | 0.163 | -0.007 | 0.053 |
| 167 | lbs, goal, pounds, hit, weighed, journey, scale, gw, 200, motivation, cico, january, plateau, loss journey, sw, cw | 0.020 | 0.02 | 0.005 | 0.036 | 0.044 | <0.001 | 0.034 | 0.054 | -0.035 | 0.136 | -0.072 | 0.002 | 0.094 | <0.001 | 0.065 | 0.123 |
| 168 | sex, women, girls, girl, porn, sexual, men, dick, drive, woman, male, gay, horny, date, female, libido | -0.018 | 0.05 | -0.033 | -0.002 | 0.007 | 0.259 | -0.004 | 0.017 | -0.059 | 0.008 | -0.096 | -0.022 | 0.033 | 0.045 | 0.003 | 0.062 |
| 169 | cravings, craving, strong, urge, thoughts, brain, fight, urges, struggling, fighting, voice, telling, intense, resist, trigger, posted | -0.020 | 0.03 | -0.035 | -0.004 | 0.046 | <0.001 | 0.036 | 0.056 | -0.038 | 0.099 | -0.075 | -0.001 | 0.130 | <0.001 | 0.101 | 0.160 |
| 170 | stress, anxiety, deal, feelings, emotions, coping, problems, depression, emotional, issues, dealing, cope, anger, struggling, angry, therapy | -0.001 | 0.96 | -0.016 | 0.015 | 0.065 | <0.001 | 0.055 | 0.075 | 0.003 | 0.937 | -0.034 | 0.040 | 0.119 | <0.001 | 0.090 | 0.149 |
| 171 | water, bottle, liquid, white, bag, powder, glass, plastic, solution, paper, inside, dry, filter, black, method, pipe | 0.026 | 0.00 | 0.010 | 0.041 | -0.025 | <0.001 | -0.036 | -0.015 | -0.004 | 0.873 | -0.041 | 0.033 | -0.115 | <0.001 | -0.144 | -0.086 |
| 172 | stop, problem, tired, wife, family, drunk, husband, scared, sick, kids, alcoholic, control, ready, bottle, wine, cycle | -0.058 | <0.001 | -0.073 | -0.042 | 0.065 | <0.001 | 0.055 | 0.076 | -0.056 | 0.013 | -0.092 | -0.019 | 0.118 | <0.001 | 0.088 | 0.147 |
| 173 | i'll, i'd, that's, can't, haven't, didn't, won't, there's, probably, isn't, doesn't, they're, wasn't, wouldn't, what's, it'll | -0.035 | <0.001 | -0.051 | -0.020 | 0.002 | 0.788 | -0.009 | 0.012 | -0.041 | 0.065 | -0.078 | -0.004 | 0.009 | 0.601 | -0.020 | 0.039 |
| 174 | aa, meeting, sober, meetings, recovery, sobriety, group, sponsor, program, steps, support, alcoholic, step, smart, power, relapse | -0.011 | 0.21 | -0.027 | 0.004 | 0.005 | 0.393 | -0.005 | 0.015 | 0.044 | 0.053 | 0.007 | 0.080 | 0.053 | <0.001 | 0.023 | 0.082 |
| 175 | pain, surgery, injury, doctor, knee, broke, ankle, recovery, leg, hospital, accident, broken, doctors, injured, foot, injuries | 0.000 | 0.98 | -0.015 | 0.016 | 0.030 | <0.001 | 0.020 | 0.040 | -0.008 | 0.758 | -0.045 | 0.029 | 0.068 | <0.001 | 0.038 | 0.097 |
| 176 | high, weed, edibles, thc, smoke, dab, pen, edible, cart, smoked, tolerance, carts, oil, marijuana, cbd, hits | 0.041 | <0.001 | 0.025 | 0.056 | -0.036 | <0.001 | -0.046 | -0.026 | -0.043 | 0.057 | -0.080 | -0.006 | 0.003 | 0.881 | -0.027 | 0.033 |
| 177 | things, fun, enjoy, bored, hobbies, productive, focus, boring, activities, spend, list, boredom, busy, play, hobby, games | -0.001 | 0.92 | -0.017 | 0.014 | 0.043 | <0.001 | 0.033 | 0.053 | -0.048 | 0.034 | -0.085 | -0.011 | 0.091 | <0.001 | 0.061 | 0.120 |
| 178 | drunk, remember, shame, drank, hurt, stupid, ashamed, blacked out, guilt, embarrassing, horrible, embarrassed, regret, blackout, black, ruined | -0.043 | <0.001 | -0.059 | -0.028 | 0.047 | <0.001 | 0.037 | 0.057 | -0.058 | 0.008 | -0.095 | -0.021 | 0.112 | <0.001 | 0.082 | 0.141 |
| 179 | cold, hot, teeth, hands, warm, mouth, face, sweat, skin, shower, eyes, dry, outside, red, heat, hair | -0.004 | 0.71 | -0.019 | 0.012 | -0.017 | 0.002 | -0.027 | -0.007 | -0.017 | 0.506 | -0.054 | 0.020 | 0.016 | 0.353 | -0.014 | 0.045 |
| 180 | smoking, smoke, quit, cigarette, cigarettes, pack, smoked, free, smoker, quitting, cravings, nicotine, smell, craving, cigs, smokes | -0.027 | 0.00 | -0.042 | -0.011 | 0.005 | 0.354 | -0.005 | 0.015 | -0.047 | 0.040 | -0.083 | -0.010 | 0.140 | <0.001 | 0.111 | 0.169 |
| 181 | love, yourself, world, loved, god, truly, wish, heart, deserve, choose, choice, beautiful, fight, power, trust, chance | -0.036 | <0.001 | -0.051 | -0.020 | 0.054 | <0.001 | 0.044 | 0.064 | -0.029 | 0.219 | -0.065 | 0.008 | 0.087 | <0.001 | 0.058 | 0.117 |
| 182 | fat, body, look, stomach, belly, legs, arms, waist, skinny, thighs, muscle, face, chest, rid, area, inches | 0.033 | <0.001 | 0.018 | 0.049 | -0.003 | 0.579 | -0.013 | 0.007 | 0.044 | 0.051 | 0.007 | 0.081 | 0.003 | 0.875 | -0.026 | 0.033 |
| 183 | vaccine, vaccines, study, health, disease, risk, article, studies, children, autism, cancer, research, science, news, evidence, vaccination | -0.018 | 0.04 | -0.034 | -0.003 | -0.007 | 0.199 | -0.017 | 0.003 | 0.025 | 0.293 | -0.012 | 0.062 | -0.061 | <0.001 | -0.090 | -0.031 |
| 184 | eat, lose, eating, healthy, diet, food, exercise, motivation, overweight, habits, unhealthy, healthier, foods, fast food, meals, junk food | 0.046 | <0.001 | 0.030 | 0.061 | 0.060 | <0.001 | 0.050 | 0.070 | -0.034 | 0.137 | -0.071 | 0.003 | 0.123 | <0.001 | 0.093 | 0.152 |
| 185 | took, felt, hours, did, feeling, hour, nothing, ago, half, later, didnt, another, yesterday, normal, minutes, effects | -0.003 | 0.73 | -0.019 | 0.012 | -0.046 | <0.001 | -0.056 | -0.036 | -0.029 | 0.215 | -0.066 | 0.008 | 0.084 | <0.001 | 0.055 | 0.114 |
| 186 | work, job, working, boss, jobs, worked, office, shift, company, career, stress, fired, position, stressful, business, manager | -0.035 | <0.001 | -0.050 | -0.019 | 0.003 | 0.579 | -0.007 | 0.013 | -0.021 | 0.399 | -0.058 | 0.016 | 0.000 | 0.984 | -0.029 | 0.030 |
| 187 | high, effects, experience, euphoria, euphoric, effect, dose, amazing, intense, strong, similar, extremely, tolerance, doses, gives, music | 0.016 | 0.07 | 0.001 | 0.032 | -0.023 | <0.001 | -0.034 | -0.013 | -0.069 | 0.002 | -0.105 | -0.032 | 0.057 | <0.001 | 0.027 | 0.086 |
| 188 | felt, were, trip, friend, decided, music, looked, room, hour, tripping, kept, talking, minutes, walk, outside, laughing | -0.025 | 0.00 | -0.041 | -0.010 | -0.062 | <0.001 | -0.072 | -0.052 | -0.084 | <0.001 | -0.121 | -0.047 | 0.103 | <0.001 | 0.073 | 0.132 |
| 189 | meth, heroin, drugs, drug, clean, using, crack, addict, dope, coke, iv, addicted, cocaine, shot, crystal, fentanyl | -0.029 | <0.001 | -0.044 | -0.013 | -0.001 | 0.861 | -0.011 | 0.009 | 0.012 | 0.634 | -0.025 | 0.049 | 0.038 | 0.019 | 0.008 | 0.067 |
| 190 | eyes, hallucinations, seeing, saw, visuals, weird, vision, room, looked, light, intense, moving, visual, black, dark, eye | 0.000 | 0.99 | -0.016 | 0.015 | -0.040 | <0.001 | -0.050 | -0.030 | -0.067 | 0.002 | -0.104 | -0.030 | 0.024 | 0.151 | -0.006 | 0.053 |
| 191 | didn't, started, did, felt, never, wanted, wasn't, went, couldn't, thought, knew, i'd, stopped, became, anymore, that's | -0.048 | <0.001 | -0.063 | -0.032 | -0.002 | 0.775 | -0.012 | 0.008 | -0.053 | 0.021 | -0.089 | -0.016 | 0.114 | <0.001 | 0.085 | 0.144 |
| 192 | she, her, wife, girlfriend, girl, told, said, asked, together, shes, gf, she's, met, daughter, woman, relationship | -0.032 | <0.001 | -0.048 | -0.017 | 0.005 | 0.377 | -0.005 | 0.015 | -0.028 | 0.226 | -0.065 | 0.009 | 0.024 | 0.149 | -0.006 | 0.053 |
| 193 | house, dog, room, smell, clean, apartment, cat, dogs, cleaning, outside, living, inside, empty, bathroom, trash, door | 0.001 | 0.90 | -0.014 | 0.017 | -0.002 | 0.664 | -0.013 | 0.008 | -0.005 | 0.869 | -0.042 | 0.032 | -0.018 | 0.278 | -0.048 | 0.012 |
| 194 | gym, workout, home, fitness, workouts, equipment, gyms, routine, membership, weights, motivation, exercises, closed, trainer, machines, cardio | -0.009 | 0.33 | -0.025 | 0.006 | 0.023 | <0.001 | 0.013 | 0.033 | 0.022 | 0.373 | -0.015 | 0.059 | 0.065 | <0.001 | 0.036 | 0.095 |
| 195 | buy, sell, price, buying, dealer, selling, bought, sold, plug, money, cheap, prices, online, dealers, street, guy | -0.014 | 0.12 | -0.029 | 0.002 | -0.032 | <0.001 | -0.042 | -0.022 | -0.009 | 0.756 | -0.046 | 0.028 | -0.090 | <0.001 | -0.119 | -0.060 |
| 196 | survey, research, study, information, uk, questions, interested, project, complete, link, university, fill, name, student, anonymous, provide | 0.006 | 0.54 | -0.010 | 0.021 | -0.002 | 0.781 | -0.012 | 0.008 | 0.027 | 0.248 | -0.010 | 0.064 | -0.035 | 0.031 | -0.064 | -0.005 |
| 197 | flight, live, city, vacation, bring, country, hotel, plane, airport, flying, travel, traveling, train, moved, town, area | -0.065 | <0.001 | -0.081 | -0.050 | -0.073 | <0.001 | -0.083 | -0.063 | -0.166 | <0.001 | -0.201 | -0.129 | -0.053 | <0.001 | -0.082 | -0.023 |
| 198 | shoes, wear, pair, helmet, shoe, boots, recommendations, jacket, feet, wearing, shorts, cycling, pedals, winter, boot, foot | -0.018 | 0.04 | -0.034 | -0.003 | -0.016 | 0.003 | -0.026 | -0.006 | 0.043 | 0.053 | 0.006 | 0.080 | -0.116 | <0.001 | -0.145 | -0.087 |
| 199 | coil, juice, coils, tank, cotton, pod, vape, burnt, taste, issue, leaking, hits, dry, liquid, burning, air | 0.016 | 0.08 | 0.000 | 0.031 | -0.017 | 0.001 | -0.027 | -0.007 | 0.047 | 0.040 | 0.010 | 0.084 | -0.120 | <0.001 | -0.150 | -0.091 |

Supplementary Table S3: Top posts from prevalent topics belonging to diet broader group pre-pandemic

| **Subreddit** | **Post** | **Pre/During pandemic** | **Broader group** | **Topic ID** | **Topic** |
| --- | --- | --- | --- | --- | --- |
| loseit | how accurate is my apple watch for calories burned? | Pre-Pandemic | Diet | 30 | Monitoring calories |
| loseit | Good calorie calculators/counters online? | Pre-Pandemic | Diet | 30 | Monitoring calories |
| loseit | Best websites that calculate calories burned? | Pre-Pandemic | Diet | 30 | Monitoring calories |
| loseit | flat belly fix burn your belly fat in 2 weeks | Pre-Pandemic | Diet | 28 | Losing body fat |
| EatCheapAndHealthy | weight loss exercises Effective Fat Burning Exercises | Pre-Pandemic | Diet | 28 | Losing body fat |
| loseit | red tea detox burns your belly fat | Pre-Pandemic | Diet | 28 | Losing body fat |
| diet | paleo diet for beginners - paleo diet benefits | Pre-Pandemic | Diet | 36 | Nutrition |
| loseit | Custom Keto Diet - Personalized Meal Plans | Pre-Pandemic | Diet | 36 | Nutrition |
| diet | Diet no carbs no sugar or Diet without sugar and carbohydrates | Pre-Pandemic | Diet | 36 | Nutrition |
| diet | Some help would be appreciated small issue Hi Reddit Last week I got my results from a blood test I did cholesterol was high (presumably due to meat) What I'd like are some tips on what I could substitute meat with. Some generally plant-based recipes are also appreciated :) Note that I'd be pretty happy if I lost 1/2 kg/week I exercise twice a week for about 2h (jogging) 78kg weight 176cm height healthy | Pre-Pandemic | Diet | 41 | Physical fitness |
| loseit | What is a healthy daily caloric deficit? Im trying to figure out how much I should eat when working out strenuously yet actively trying to lose weight. I dont really know how to factor in exercise and I want to eat enough to satisfy my bodys needs yet lose weight but not unhealthily. Basically how much is too much for a caloric deficit? TIA | Pre-Pandemic | Diet | 41 | Physical fitness |
| loseit | After years of saying Ill exercise tomorow I finally got my ass to the gym and I have never been proud of myself until now. | Pre-Pandemic | Diet | 41 | Physical fitness |
| loseit | Eating 1200 calories a day + cardio not losing weight | Pre-Pandemic | Diet | 82 | Weight loss |
| loseit | Not losing weight on 1200 cals what to do? | Pre-Pandemic | Diet | 82 | Weight loss |
| diet | Is there a diet that will help me get a flatter stomach? I find that most of my weight goes directly to my stomach. Is there any way around this through my diet? Thank you! | Pre-Pandemic | Diet | 82 | Weight loss |

Supplementary Table S4: Top posts from prevalent topics belonging to diet broader group during pandemic

| **Subreddit** | **Post** | **Pre/During pandemic** | **Broader group** | **Topic ID** | **Topic** |
| --- | --- | --- | --- | --- | --- |
| loseit | Calorie calculators say I burned 700 calories my bike says I burned 350. Why? | During Pandemic | Diet | 30 | Monitoring calories |
| EatCheapAndHealthy | I dont know how to get enough calories on my budget. What are the cheapest most calorie dense foods? COVID has hit me pretty hard and Ive been working with an impossibly tight budget these last few months. I can only afford 1-2meals a day what can I eat very little of but still be able to stop losing/put back on some weight? Thanks in advance. | During Pandemic | Diet | 30 | Monitoring calories |
| GettingShredded | Looking for help on getting past plateau. SW: 215 CW: 193 GW: 169...stalled 2 months at 1700 cals. Was a leaner and strong 200 years ago but with no access to strong lifts due to COVID struggling with how to burn fat and want to cut for health reasons. Appreciate any support! | During Pandemic | Diet | 28 | Losing body fat |
| diet | Do Fat burners and weight loss supplements work? | During Pandemic | Diet | 28 | Losing body fat |
| diet | Vegan Protein Sources - High Protein Vegan | During Pandemic | Diet | 36 | Nutrition |
| GYM | Gym Gains advice Im a 20 yrd old male 6 foot 173 lbs. I started lifting last week after not hitting the gym since March due to Covid. I have been doing a back/bi chest/tri legs shoulders split. For nutrition I am trying to eat chicken and rice every night for dinner. I am also drinking protein powder every single day. Any advice on nutrition specifically? Easy meals I can easy that have lots of protein and calories? | During Pandemic | Diet | 36 | Nutrition |
| diet | My workouts arent working anymore? I still cant go to gyms and stuff (because of the Covid lockdown). I never get sore from my home workouts anymore. Ive tried to do harder ones and I still never get sore or feel like its working at all. Any suggestions of what I could do? | During Pandemic | Diet | 41 | Physical fitness |
| GYM | Need some help about workout at home I stopped gym and locked myself due to COVID-19 outbreak. After 2 weeks of non-exercise i decided to start workout at home but right now i dont know which exercises would fit to my current standards. I would like to read suggestions by veterans/professionals. (NOTE: i don't have any equipment at home about my standards my weight is 70k and my height is 180cm and i recently started lifting after successful weight loss). | During Pandemic | Diet | 41 | Physical fitness |
| diet | Am I losing muscle mass?? So before quarantine I was consistently going to the gym. I wasnt losing weight because I was shedding fat and simultaneously putting on muscle so it sort of evened out. Now that Im not able to workout as consistently I have lost about 8 pounds but havent seen any loss of fat. Could this be a loss of muscle mass and is there any way to prevent this through my diet. I really hope that all those hours havent gone to waste. Also I will say I have been eating less since I came back home from college due to Covid-19 but I dont think its enough for this big of a weight loss. (Btw Im a woman and I assure you am not losing weight because of covid) | During Pandemic | Diet | 82 | Weight loss |
| GYM | Advice for starting out. Im 511 203.3 15 year old. Im trying to lose about 20 pounds and start gain muscle instead of fat. I have very strong legs and really weak arms. I need to be ready for football season in March (its late start because of COVID.)What should I do to lose the weight in a reasonable time and gain muscle? Also should I continue counting my calories or try another method to lose weight I was thinking about fasting. | During Pandemic | Diet | 82 | Weight loss |

Supplementary Table S5: Top posts from prevalent topics belonging to physical activity broader group pre-pandemic

| **Subreddit** | **Post** | **Pre/During pandemic** | **Broader group** | **Topic ID** | **Topic** |
| --- | --- | --- | --- | --- | --- |
| bicycling | Biking in Paris: Bike Lanes Bikeshare and Bike Culture in France | Pre-Pandemic | Physical Activity | 141 | City biking |
| bicycling | Is this weird? Is it weird that on long bike trips with my friends I like to talk to them on the phone?What we do is that we all sync up our phones to our headphones/airpods so that we can all chat on our way to the mall or wherever we wanna go.Doing this works somewhat well but most of the time the mic picks up a lot of wind. But that's not what this post is about.Do you guys thinks it's weird to do calls when biking or nah? | Pre-Pandemic | Physical Activity | 141 | City biking |
| bicycling | 30 Days of Biking. Starts in less than a week. Who else is participating? | Pre-Pandemic | Physical Activity | 141 | City biking |
| backpacking | 24 Top-rated tourist attractions in munich germany | Pre-Pandemic | Physical Activity | 35 | International travel |
| backpacking | It's always a nice idea to have a homestay to come back to in Bali. This is how we had to do our laundry though! | Pre-Pandemic | Physical Activity | 35 | International travel |
| backpacking | Travelling From Ao Nang To Railay Thailand and seeing hidden caves (no tourists there!) and the quieter beaches | Pre-Pandemic | Physical Activity | 35 | International travel |
| backpacking | Backpacking central america and Colombia for a few months any advice? March 2020 I'm flying to Colombia and will travel nothwards to Mexico in a few months time. I'll be togheter with my gf. This is my second big backpacking trip and her first. My question is:Do you have any must sees or highlights in Col of CA that we can't miss. Also any highlights that are easy to reach but not too touristy?We are interested in nature and sporting activities (hiking biking etc) | Pre-Pandemic | Physical Activity | 79 | Backpacking/Hiking |
| trailrunning | Recommendations for backpacking Europe? Middle/West My route will be Russia-Belarus-Poland- Czech Republic-Slovakia-Austria-Hungary-Slovenia-maybe Croatia and definitely Romania. As of now I have 2-2.5 months to travel. Is it gonna be enough?So please share your travels ideas and recommendations on these places. I am interested in live music outdoors (hiking biking etc. and actually trying to find a nice route to hike maybe 2-3 days (beginner level) around Austria and Slovenia) skateboarding and I am not much of a partier though I do like a nice pub or some techno once in a while. I would rather avoid the big cities even though I will go there for skateboarding otherwise I love the countryside. | Pre-Pandemic | Physical Activity | 79 | Backpacking/Hiking |
| backpacking | Hitchhiking from Finland to Australia Hey Reddit!So I am on a mission to hitchhike from Finland to Australia. Atm I'm in southern Cambodia where I'll stay for the next few weeks before heading to Thailand.Around 2 months back I made a small post about my trip to a Finnish traveling group and I was really surprised how much attention it got in the end. I've been reading Reddit now for years but never really wrote anything here so I thought it would be cool idea to host a small AMA if there are people who are interested to know more about budget backpacking and hitchhiking.My Instagram for random pictures: FindheikkiCheers | Pre-Pandemic | Physical Activity | 79 | Backpacking/Hiking |

Supplementary Table S6: Top posts from prevalent topics belonging to physical activity broader group during pandemic

| **Subreddit** | **Post** | **Pre/During pandemic** | **Broader group** | **Topic ID** | **Topic** |
| --- | --- | --- | --- | --- | --- |
| bicycling | Completely new to cycling. Advice on a bike? Hi everyone First time here. I'm completely new to bicycling...I used to be a runner (pre-covid). I got really sick over the summer gained a bit of weight and I thought that bicycling would be a good exercise alternative that might be easier on my joints. To that end I'm looking for advice on an affordable bike for a 6'0 man. I'm mostly planning on biking along roads rather than mountains or off-road. If anyone could point me towards some affordable starter bikes I would really appreciate it!" | During Pandemic | Physical Activity | 141 | City biking |
| bicycling | What Tips would you give to a new Biker? What would be the best tips that you have been given or learned over the years biking?Hello from Mumbai India. Just started Biking Main Objective to lose weight gained due to less outdooractivity during this year. Already had 25% body fat Pre-Covid and was looking for a new hobby as well. | During Pandemic | Physical Activity | 141 | City biking |
| bicycling | Wanting to get into bicycling Im 28 and trying to get in shape and always loved riding bikes. I want to get a bike that I can ride on the road and also go mountain biking with occasionally if thats a thing. Does anyone have a recommendation for a beginners bike under 1300? And what are some essentials to have besides the bike itself to get started.Also am I too heavy atm to even start riding? Im around 260 atm. | During Pandemic | Physical Activity | 141 | City biking |
| backpacking | Is international travel going to even be possible when all the airlines open? Is this a good time to get a dog? Apparently after 9/11 it took 3 years for airlines to pick up in America. I just got a remote career and was planning to backpack across the world - but it seems like that might not be an option anytime soon. Ive been holding off getting a dog for this reason so maybe now is the time (hoping Id have more money in the future for boarding or more people that the dog would be comfortable around if I did need to travel) | During Pandemic | Physical Activity | 35 | International travel |
| backpacking | To travel or not to travel? Hey all! I did a 6-month backpacking trip in SE Asia last year and have a couple of months off until life gets crazy again. I was planning this before all of this started so I'm pretty bummed. Am I completely off base to think that I can still go to Indonesia in June? Can someone give me a general rundown of how it is right now considering COVID? Is everything still accessible or is my paranoia getting the best of me. Thanks in advance! | During Pandemic | Physical Activity | 35 | International travel |
| backpacking | Anyone Backpacking during COVID? Hi I'm 21 years old living in London working part time. I have been meaning to travel since I got back from India in March but have been waiting for COVID to die down. Unfortunately that's not going too well. I did go on a little roadtrip through Germany but other than that not much. I was thinking to just go New Zealand for a couple months using [workaway.info](https://workaway.info) (or anywhere that'll let me tbh lol). I was wondering has anyone planned any trips recently if so where to and any tips.Thanks :) | During Pandemic | Physical Activity | 35 | International travel |
| backpacking | Im wondering if there are any 4-person tents suitable for backpacking Looking for one that could withhold a storm lightweightetc... but most places recommend 1-2 person tents for backpacking due to weight. I will be looking to hike a good bit but most of if not all our travels will be with 4 people. Thanks! | During Pandemic | Physical Activity | 79 | Backpacking/Hiking |
| trailrunning | Trail running vacations? Day dreaming about traveling again and wanted to hear suggestions for trail running destinations? I try to center my traveling around my sports" - mountain biking backpacking and trail running." | During Pandemic | Physical Activity | 79 | Backpacking/Hiking |
| trailrunning | Trail shoes or heavy boots Iceland trail Hello I'm going to do the Iceland trail with some friends next summer and i was wondering what shoes do you all recommend for something like that. Are heavy waterproof boots the best option or are lightweight breathing shoes better?It'll be my first big trail but i regularly do mountain biking in the woods so my feet are somewhat trained. | During Pandemic | Physical Activity | 79 | Backpacking/Hiking |

Supplementary Table S7: Top posts from prevalent topics belonging to substance use broader group pre-pandemic

| **Subreddit** | **Post** | **Pre/During pandemic** | **Broader group** | **Topic ID** | **Topic** |
| --- | --- | --- | --- | --- | --- |
| Drugs | 30mg adderall 1mg xanax and 0.5mg klonopin | Pre-Pandemic | Substance Use | 40 | Benzodiazepines |
| Drugs | Etiz and xan Is it safe to mix etizolam and Xanax? | Pre-Pandemic | Substance Use | 40 | Benzodiazepines |
| Drugs | How to reverse effects of DXM Polistirex? | Pre-Pandemic | Substance Use | 67 | Nootropics |
| Drugs | DXM with Piracetam 300mg DXM with 25g Piracetam. What should i expect? | Pre-Pandemic | Substance Use | 67 | Nootropics |
| Drugs | Gabapentin withdrawal Is gabapentin withdrawal as bad as benzo withdrawal is it something that can last for months/years like benzo withdrawal? | Pre-Pandemic | Substance Use | 87 | Withdrawal symptoms |
| alcoholism | If your withdrawal symptoms stop and you feel fine are you still at risk for developing more severe symptoms such as seizures and DTs? nan | Pre-Pandemic | Substance Use | 87 | Withdrawal symptoms |
| stopdrinking | Weening off Alcohol Any tips on weening off alcohol to avoid withdrawals. | Pre-Pandemic | Substance Use | 87 | Withdrawal symptoms |
| alcoholism | Non alcohol beer with Campral?? Is it safe to drink non alcoholic beer while taking Campral? Seems that Heineken is the only 0.0 % non alcoholic beer | Pre-Pandemic | Substance Use | 111 | Beer and Wine |
| alcoholism | Pennsylvania Brewery Debuts IPA Infused with Cannabis Terpenes | Pre-Pandemic | Substance Use | 111 | Beer and Wine |
| alcoholism | Alcoholism and Alcohol Abuse: Signs Risks and Side Effects | Pre-Pandemic | Substance Use | 132 | Problems due to alcoholism |
| stopdrinking | After 18 days without my brain is asking only alcohol alcohol alcohol alcohol | Pre-Pandemic | Substance Use | 132 | Problems due to alcoholism |
| addiction | Drug and Alcohol Detox - The First Step to Sobriety [alcohol detoxification treatment] [alcohol rehabilitation centers] [inpatient drug rehab] [holistic treatment] [drug intervention program] | Pre-Pandemic | Substance Use | 49 | Rehab/Therapy |
| Drugs | Couples Drug Rehab \| Couples Rehab Detox Treatment Centers | Pre-Pandemic | Substance Use | 49 | Rehab/Therapy |
| stopdrinking | NYC Peeps-Listen Bar Sober Halloween Karaoke Listen Bar is a NA "bar" in the city and they are hosting a sober karaoke party on Halloween their mixologists make the coolest "mock" tails. Should be fun! | Pre-Pandemic | Substance Use | 154 | Social drinking |
| stopdrinking | Went to a concert then a bar then a house party | Pre-Pandemic | Substance Use | 154 | Social drinking |
| stopdrinking | How do you cope with a bad day? Im fighting an ear infection and am under enormous stress at work. What are your healthy coping mechanisms? | Pre-Pandemic | Substance Use | 170 | Stress/Anxiety |
| stopdrinking | What is it about alcohol that makes us think it cures loneliness boredom or depression. I guess Im trying to understand coping mechanisms. Trying to understand how you can recognize that a coping mechanism is not very effective but yet keep using it. Thoughts? | Pre-Pandemic | Substance Use | 170 | Stress/Anxiety |
| addiction | Check out my youtube video discussing depression self hate the self fulfilling prophecy and coping mechanisms | Pre-Pandemic | Substance Use | 170 | Stress/Anxiety |
| Drugs | buy morphine buy morphine online buy morphine australia | Pre-Pandemic | Substance Use | 155 | Opioids |

Supplementary Table S8: Top posts from prevalent topics belonging to substance use broader group during pandemic

| **Subreddit** | **Post** | **Pre/During pandemic** | **Broader group** | **Topic ID** | **Topic** |
| --- | --- | --- | --- | --- | --- |
| Drugs | Ativan (Lorazepam) 2mg - Cryptomedstore | During Pandemic | Substance Use | 40 | Benzodiazepines |
| Drugs | Mixing benzos Can multiple benzos be mixed together - 2mg xanax 10mg diazepam and some beer the Xanax may have etizolam | During Pandemic | Substance Use | 40 | Benzodiazepines |
| Drugs | Does anyone else enjoy Benadryl/DPH highs? | During Pandemic | Substance Use | 67 | Nootropics |
| Drugs | Dextromethorphan hydrobromide light dose just for fun? | During Pandemic | Substance Use | 67 | Nootropics |
| addiction | Withdrawal Whats great to take for cravings and withdrawal symptoms? | During Pandemic | Substance Use | 87 | Withdrawal symptoms |
| Drugs | Which is worse benzo withdrawals or opioid withdrawals | During Pandemic | Substance Use | 87 | Withdrawal symptoms |
| Drugs | What is worse: Kratom withdrawal or Benzo withdrawal? | During Pandemic | Substance Use | 87 | Withdrawal symptoms |
| stopdrinking | Are my symptoms of Alcohol Withdrawals normal? | During Pandemic | Substance Use | 87 | Withdrawal symptoms |
| stopdrinking | Alcohol withdrawal Has anybody ever used phenibut for WD symptoms? And if so whats been your experience when withdrawing? | During Pandemic | Substance Use | 87 | Withdrawal symptoms |
| stopdrinking | Just poured 20 litres of my homebrew beer down the sink | During Pandemic | Substance Use | 111 | Beer and Wine |
| stopdrinking | Poured myself some wine Then threw it down the sink. Had ice cream instead. IWNDWYT | During Pandemic | Substance Use | 111 | Beer and Wine |
| alcoholism | Any moderate drinkers give up drinking entirely? | During Pandemic | Substance Use | 132 | Problems due to alcoholism |
| alcoholism | Alcohol Problems - 23 Years Of Drinking Alcohol - Pain/Weak/Blackouts | During Pandemic | Substance Use | 132 | Problems due to alcoholism |
| addiction | Addiction Recovery Hope Help &amp; Rehabilitation - Serenity Vista \| International Addiction Treatment Center \| Alcoholism and Drug Rehab | During Pandemic | Substance Use | 49 | Rehab/Therapy |
| addiction | What should I expect in detox IOP PHP residential Sober Living | During Pandemic | Substance Use | 49 | Rehab/Therapy |
| stopdrinking | Any bartenders here? For real how do we do it ? | During Pandemic | Substance Use | 154 | Social Drinking |
| alcoholism | Bartenders Are bartenders enablers? Im not blaming them...but after losing my fianc to alcoholism I could never be a bartender... | During Pandemic | Substance Use | 154 | Social Drinking |
| alcoholism | Coping Mechanisms I tend to use alcohol as a coping mechanism. Once quitting I have pretty much just gone to bed early so Im not tempted to drink. What are some other healthy coping mechanisms for stressful situations or days which is my main trigger? | During Pandemic | Substance Use | 170 | Stress/Anxiety |
| alcoholism | Does consuming alcohol as a coping mechanism count as dependence? | During Pandemic | Substance Use | 170 | Stress/Anxiety |
| Marijuana | Should I continue using marijuana as medicine/coping mechanism? | During Pandemic | Substance Use | 170 | Stress/Anxiety |
| stopdrinking | Day 55 How's everyone coping with the lock down?George | During Pandemic | Substance Use | 170 | Stress/Anxiety |
| Drugs | buy morphine buy morphine online buy morphine usa | During Pandemic | Substance Use | 155 | Opioids |

Supplementary Table S9: Top posts from prevalent topics belonging to smoking broader group pre-pandemic

| **Subreddit** | **Post** | **Pre/During pandemic** | **Broader group** | **Topic ID** | **Topic** |
| --- | --- | --- | --- | --- | --- |
| stopsmoking | Will one cigarette trigger withdrawal symptoms again? | Pre-Pandemic | Smoking | 87 | Withdrawal symptoms |
| electronic_cigarette | Sore throat and cough after vaping. | Pre-Pandemic | Smoking | 160 | Pulmonary sickness |
| electronic_cigarette | Starting to feel shortness of breath and something in lungs while breathing (maybe vaping...) | Pre-Pandemic | Smoking | 160 | Pulmonary sickness |
| electronic_cigarette | Cigarette free since a month wish me luck on quitting | Pre-Pandemic | Smoking | 180 | Quit smoking |
| quittingsmoking | NOT ANOTHER PUFF NO MATTER WHAT! | Pre-Pandemic | Smoking | 180 | Quit smoking |
| stopsmoking | 45 days without a toxic puff of cigarette smoke! | Pre-Pandemic | Smoking | 180 | Quit smoking |
| quittingsmoking | Nicotine Gum Only? Anyone successfully quit using the gum only? | Pre-Pandemic | Smoking | 130 | Other sources of nicotine |
| stopsmoking | Nicorette should make tobacco flavored gum. Mint gum tastes terrible with a glass of scotch. nan | Pre-Pandemic | Smoking | 130 | Other sources of nicotine |
| smokingcessation | Hi! STOP CRAVING Hello! I'm Lucas from Argentina and I don't speak English. I am using the google translator to write. Tomorrow I quit smoking. I have smoked for 15 years and lately I smoke 20 cigarettes a day. My strategy is not to fantasize about smoking. It is clear to me that if I think about smoking it is almost the same as smoking in my brain. So the addiction is activated and therefore the CRAVING. Tomorrow I start I will inform you if I can control the craving. Regards! | Pre-Pandemic | Smoking | 169 | Craving to smoke |
| stopsmoking | Day 3 off cigarettes with intense cravings! Been trying to keep busy by eating tictacs chewing nicotine gum. Will the cravings ever go away? | Pre-Pandemic | Smoking | 169 | Craving to smoke |

Supplementary Table S10: Top posts from prevalent topics belonging to smoking broader group during pandemic

| **Subreddit** | **Post** | **Pre/During pandemic** | **Broader group** | **Topic ID** | **Topic** |
| --- | --- | --- | --- | --- | --- |
| smokingcessation | This is helping me fight the withdrawal symptoms. | Pre-Pandemic | Smoking | 87 | Withdrawal symptoms |
| quittingsmoking | Shortness of breath Going on the 4 month mark. Does anyone else notice an incline in shortness of breath? | Pre-Pandemic | Smoking | 160 | Pulmonary sickness |
| stopsmoking | How to get rid of the mucus from my Lungs Sinuses and Throat after stop Smoking? I do not suffer from Coughs and still feeling shortness of breath. I quitted smoking since 15 Days. | Pre-Pandemic | Smoking | 160 | Pulmonary sickness |
| stopsmoking | New ex smoker I just smoked my last cigarette. Wish me luck | Pre-Pandemic | Smoking | 180 | Quit smoking |
| stopsmoking | Six months cigarette and nicotine free ! | Pre-Pandemic | Smoking | 180 | Quit smoking |
| stopsmoking | I quit smoking! Not another puff no matter what! | Pre-Pandemic | Smoking | 180 | Quit smoking |
| stopsmoking | Does switching from smoking cigarettes to using snus pouches count as quitting? | Pre-Pandemic | Smoking | 130 | Other sources of nicotine |
| stopsmoking | Is snus or nicotin gums a better alternative for smoking? | Pre-Pandemic | Smoking | 130 | Other sources of nicotine |
| quittingsmoking | Relapsed after 1 d 14 hrs I feel so fucking weak. The craving wasnt even that bad I just had an opportunity to smoke. Actually I was craving more for nicotine spray that Ive used as replacement but I decided to smoke instead. Yes I am addicted to smoking but I still dont understand why I cant stop | Pre-Pandemic | Smoking | 169 | Craving to smoke |
| quittingsmoking | Made it three weeks!! The last time I quit it I quit cold turkey and only lasted 28 days. This time I am using the patches and it really helps with cravings. I also began exercising daily. Introducing a new routine in my life has made me feel healthier and is something I never did when I smoked. | Pre-Pandemic | Smoking | 169 | Craving to smoke |

Supplementary Table S11: Interrupted Time Series (ITS) regression output for weekly count of messages

| **Broader Group** | **Response Variables** | **Coefficients** | **Standard Error** | **t-statistic** | **p-value** | **CI (0.025)** | **CI (0.975)** | **Impact % (I)** |
| --- | --- | --- | --- | --- | --- | --- | --- | --- |
| Diet | Intercept | 2247.4911 | 57.362 | 39.181 | < 0.001 | 2133.659 | 2361.324 |  |
|  | week | 1.1418 | 1.596 | 0.715 | 0.476 | -2.026 | 4.309 |  |
|  | **pandemic** | **829.3389** | **94.594** | **8.767** | **< 0.001** | **641.621** | **1017.057** | **36.90** |
|  | week*pandemic | -16.2833 | 3.646 | -4.467 | < 0.001 | -23.518 | -9.049 |  |
| Physical Activity | Intercept | 1399.1592 | 48.031 | 29.13 | < 0.001 | 1303.843 | 1494.475 |  |
|  | week | 2.0512 | 1.336 | 1.535 | 0.128 | -0.601 | 4.703 |  |
|  | **pandemic** | **807.4319** | **79.207** | **10.194** | **< 0.001** | **650.248** | **964.616** | **57.70** |
|  | week*pandemic | -18.4539 | 3.053 | -6.045 | < 0.001 | -24.511 | -12.396 |  |
| Substance Use | Intercept | 4556.6776 | 74.194 | 61.416 | < 0.001 | 4409.385 | 4703.97 |  |
|  | week | -2.2409 | 2.169 | -1.033 | 0.304 | -6.547 | 2.065 |  |
|  | **pandemic** | **-36.3443** | **120.539** | **-0.302** | **0.764** | **-275.644** | **202.956** | **-0.79** |
|  | week*pandemic | -27.6668 | 4.673 | -5.92 | < 0.001 | -36.945 | -18.389 |  |
| Smoking | Intercept | 965.2634 | 22.772 | 42.388 | < 0.001 | 920.055 | 1010.472 |  |
|  | week | -1.8137 | 0.666 | -2.724 | 0.008 | -3.135 | -0.492 |  |
|  | **pandemic** | **-11.3579** | **36.997** | **-0.307** | **0.76** | **-84.806** | **62.09** | **-1.17** |
|  | week*pandemic | -5.7944 | 1.434 | -4.04 | < 0.001 | -8.642 | -2.947 |  |

Supplementary Table S12: Interrupted Time Series (ITS) regression output for weekly new user counts

| **Broader Group** | **Response Variables** | **Coefficients** | **Standard Error** | **t-statistic** | **p-value** | **CI (0.025)** | **CI (0.975)** | **Impact % (I)** |
| --- | --- | --- | --- | --- | --- | --- | --- | --- |
| Diet | Intercept | 1033.5331 | 33.527 | 30.827 | < 0.001 | 966.999 | 1100.067 |  |
|  | week | -5.3052 | 0.964 | -5.504 | < 0.001 | -7.218 | -3.392 |  |
|  | **pandemic** | **372.7218** | **53.872** | **6.919** | **< 0.001** | **265.814** | **479.629** | **36.06** |
|  | week*pandemic | -0.5002 | 1.997 | -0.25 | 0.803 | -4.464 | 3.463 |  |
| Physical Activity | Intercept | 564.028 | 28.363 | 19.886 | < 0.001 | 507.743 | 620.313 |  |
|  | week | -3.6903 | 0.815 | -4.526 | < 0.001 | -5.308 | -2.072 |  |
|  | **pandemic** | **429.1378** | **45.574** | **9.416** | **< 0.001** | **338.698** | **519.578** | **76.08** |
|  | week*pandemic | -4.0609 | 1.69 | -2.403 | 0.018 | -7.414 | -0.708 |  |
| Substance Use | Intercept | 1600.806 | 40.714 | 39.318 | < 0.001 | 1519.979 | 1681.633 |  |
|  | week | -12.296 | 1.232 | -9.982 | < 0.001 | -14.741 | -9.851 |  |
|  | **pandemic** | **121.6379** | **64.451** | **1.887** | **0.062** | **-6.314** | **249.59** | **7.59** |
|  | week*pandemic | 2.2528 | 2.411 | 0.934 | 0.353 | -2.534 | 7.04 |  |
| Smoking | Intercept | 413.1175 | 12.368 | 33.403 | < 0.001 | 388.565 | 437.67 |  |
|  | week | -3.0884 | 0.374 | -8.254 | < 0.001 | -3.831 | -2.346 |  |
|  | **pandemic** | **37.5325** | **19.578** | **1.917** | **0.058** | **-1.335** | **76.4** | **9.08** |
|  | week*pandemic | 0.7242 | 0.732 | 0.989 | 0.325 | -0.73 | 2.178 |  |

Supplementary Table S13: Comparison of Coherence Scores across different topic sizes using Latent Dirichlet Allocation

| **Coherence Measure** | **50 Topics** | **100 Topics** | **200 Topics** |
| --- | --- | --- | --- |
| C_V | 0.5933 | 0.5768 | 0.6027 |
| C_UCI | 0.9175 | 0.9631 | 0.9520 |
| C_NPMI | 0.0991 | 0.0992 | 0.1022 |
